# Fast process-level screening of metal–organic frameworks for adsorption separation using 3D classical density functional theory

**DOI:** 10.1039/d6me00066e

**Published:** 2026-06-27

**Authors:** Marcel Granderath, Philipp Rehner, Joachim Gross, Vincent Dufour-Décieux, André Bardow

**Affiliations:** a Energy & Process Systems Engineering, Department of Mechanical and Process Engineering, ETH Zurich Tannenstrasse 3 8092 Zurich Switzerland vdufour@ethz.ch abardow@ethz.ch; b Institute of Thermodynamics and Thermal Process Engineering, University of Stuttgart Pfaffenwaldring 9 70569 Stuttgart Germany

## Abstract

Adsorption-based separation using metal–organic frameworks (MOFs) represents a promising, energy-efficient alternative to conventional separation technologies. While the vast design space of MOFs allows for precise property tuning to specific applications, the sheer scale of the design space renders exhaustive experimental testing impractical. Consequently, materials discovery relies on large-scale computational screening. Current screening workflows typically employ Grand-Canonical Monte Carlo (GCMC) simulations to predict adsorption properties; however, the high computational cost of GCMC often limits the number of materials screened. In this work, we introduce a process-level screening paradigm leveraging the computational speed of GPU-accelerated 3D classical density functional theory (cDFT). Validation against state-of-the-art GCMC simulations demonstrates that cDFT accurately predicts process key performance indicators (KPIs). The KPIs calculated *via* cDFT generally deviate by less than 5% from GCMC results while reducing computational costs by two to four orders of magnitude. Leveraging this increased efficiency, we screen the publicly available CoRE MOF 2025 database for methane/nitrogen separation using a temperature-swing adsorption process. By evaluating performance across a wide range of feed compositions, from industrial gas streams to dilute methane sources, the study identifies MOF candidates with robust performance profiles. The entire screening of over 5000 MOFs, requiring 460 000 adsorption calculations, was completed in only five days using two GPUs. The work establishes cDFT as a reliable, high-throughput approach for process-informed material discovery.

## Abstract 2

Classical density functional theory enables fast and accurate process-level screening of metal–organic frameworks for adsorption separation.

## Graphical abstract

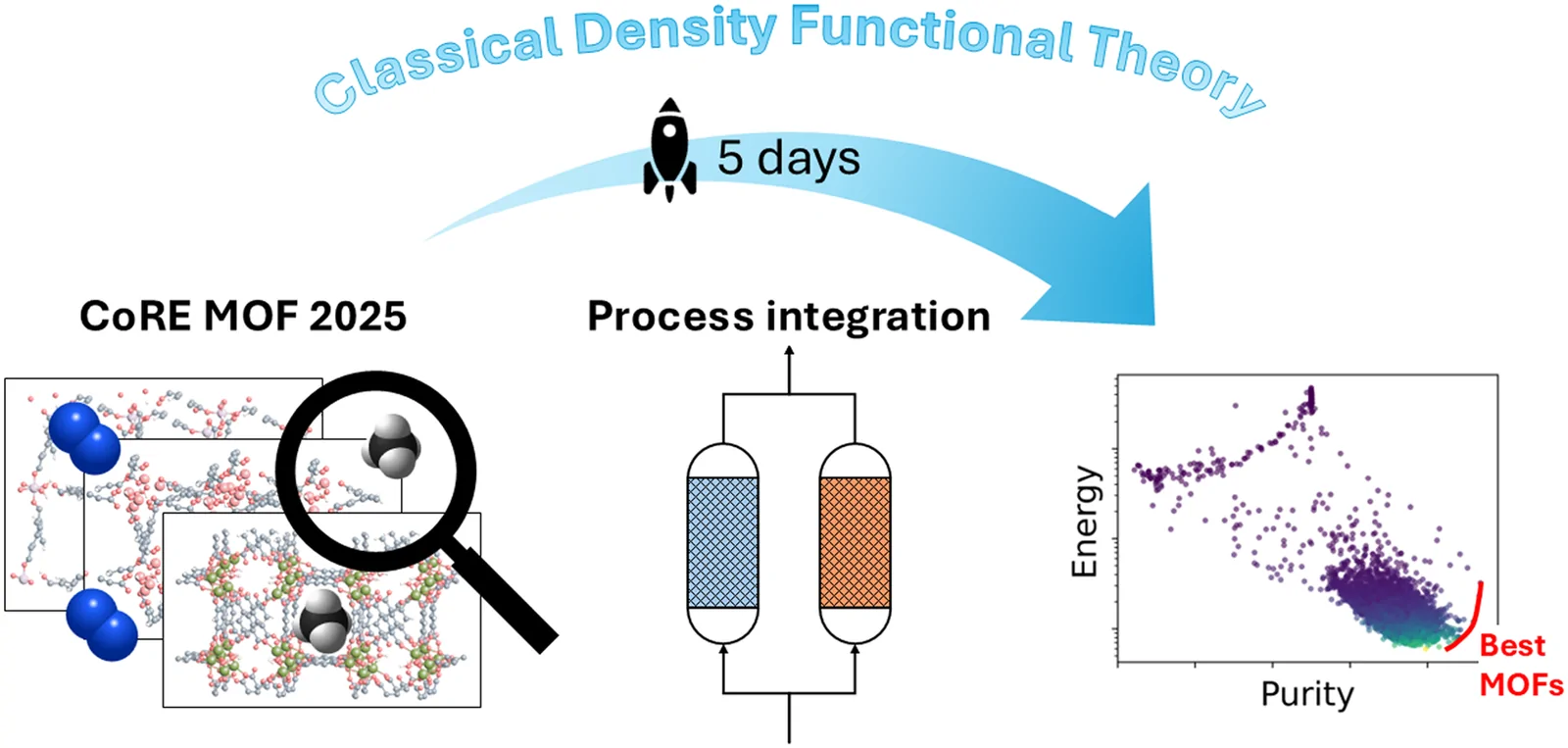

**Design, System, Application**

Adsorption-based gas separation using metal-organic frameworks (MOFs) represents an energy-efficient alternative to conventional separation technologies. The size of the MOF design space makes exhaustive experimental testing impractical. State-of-the-art molecular simulations are computationally expensive, limiting the number of materials that can be screened. In this work, we introduce a high-throughput computational screening methodology leveraging GPU-accelerated 3D classical density functional theory (cDFT). We show that cDFT accurately predicts key performance indicators while reducing computational costs by two to four orders of magnitude compared to state-of-the-art molecular simulations. Future use of this methodology can accelerate the discovery and deployment of MOFs tailored to a wide range of industrial requirements.

## Introduction

Separation processes are fundamental to the chemical industry but are also responsible for a significant fraction of its energy consumption.^1^ In recent years, energy-efficient separation processes have been explored based on adsorption on nanoporous materials, in particular metal–organic frameworks (MOFs).^2^ MOFs show exceptional tunability regarding pore geometry, size, and chemical functionality.^2^ This design flexibility has led to tens of thousands of structures being synthesized experimentally and millions more being designed computationally.^3,4^ This vast chemical design space comes with the promise of finding optimal materials with properties fine-tuned for a specific separation but also the challenge of identifying the best materials among such a large number of candidates. This large material space is intractable for experimental synthesis and characterization. Consequently, the common approaches rely on computational screening.^4–6^

Computational screenings rank the MOFs according to metrics that should capture the performance of the MOF for a specific application. These metrics are commonly material properties such as the selectivity of a MOF for an adsorbate over another one at a specific pressure and temperature.^7–9^ However, recent studies assess process-level performance and highlight that material properties do in general not correlate well with industrially relevant metrics such as energy demand, product purity and productivity.^10–12^ Screening pipelines thus need to include process simulations.^11,12^

Process simulations require adsorption properties across a broad set of temperatures, pressures, and compositions.^11,13^ Established methods such as Grand-Canonical Monte Carlo (GCMC) simulations can predict adsorption accurately.^11,13^ However, single GCMC simulations at a fixed temperature, pressure, and composition can take up to a few hours, making their direct inclusion in process simulations prohibitively expensive.^14^ To reduce the computational cost, mixture adsorption at different temperatures is usually predicted by simplified methods based on pure-component adsorption isotherms.^11,13^ Common methods are the Clausius–Clapeyron (CC) equation for temperature extrapolation and for mixture predictions using the ideal adsorbed solution theory (IAST) or the extended dual-site Langmuir model.^15,16^ Accurate extrapolation with IAST requires the evaluation of full isotherms, from the Henry regime to the saturation regime.^17^ Full adsorption isotherm evaluation with GCMC takes hours to days, limiting the number of materials in GCMC-based screenings even when using extrapolation models. Current developments therefore try to speed up GCMC computations.^18^

Recently, classical density functional theory (cDFT) has emerged as an accurate and fast method to predict adsorption properties.^14,19,20^ cDFT predictions require similar input as GCMC simulations: an adsorbent structure representation (*e.g.*, a Crystallographic Information File (CIF)), and a force-field description of the solid–gas interactions, but also a Helmholtz energy functional to describe the gas phase, typically available from bulk fluid equations of state.^21^ cDFT has been successfully validated against GCMC for a diverse range of adsorbent–adsorbate systems.^14,19,22–26^ cDFT predictions of both, adsorption isotherms and enthalpies of adsorption show almost perfect agreement with GCMC simulations for small molecules without coulombic interactions, such as methane, nitrogen, and hydrogen.^19,23^ Molecules with coulombic interactions (*e.g.*, carbon dioxide) can still be described with good agreement compared to GCMC simulations.^19^

In addition to preserving accuracy, the main advantage of cDFT is its low-cost, noise-free computations. The computational time of cDFT is two to three orders of magnitude lower than GCMC simulations when run on CPUs.^14^ Further computational speedup is achieved by leveraging GPU-accelerated automatic differentiation.^20^ Noise-free results are instrumental for performing optimizations of processes or adsorbent structures.

The accuracy and computational speed of cDFT enabled already MOF screenings for separation and storage applications.^27–29^ Guo *et al.* screen 1200 MOFs for methane/hydrogen separation with cDFT, based on the selectivity at single pressure and temperature points without evaluating the enthalpy of adsorption. Their model can only represent spherical molecules.^27^ Similarly, Fu *et al.* study the adsorption of methane in 1200 MOFs using 1D cDFT.^28^ The simplified 1D approximation of a structure requires initial fitting of molecular simulation data and cannot predict adsorption isotherms from scratch. Thus, 1D cDFT can extrapolate adsorption properties,^30^ but it is not suited for exploring new structures. Mayer *et al.* screen a database of 775 MOFs for carbon capture including process simulation; however, their study is also limited to 1D cDFT.^29^

While previous studies show small deviations across predictions of adsorption isotherms and enthalpies of adsorption by cDFT,^19,31^ the propagation of these deviations to process level KPI's has not been investigated. Further, the speed of 3D cDFT has so far not been leveraged for screenings of MOFs including process simulations.^31^

In this work, we propose a screening workflow based on 3D cDFT at the process level. For the first time, we validate cDFT for methane/nitrogen separation processes, compared to the state-of-the-art method based on GCMC simulations. The validation includes binary GCMC simulations for adsorption properties of 39 MOFs and process-level evaluation of ∼850 MOFs. After this validation, we leverage the computational speed of GPU-accelerated cDFT to conduct a large-scale process-level screening of MOFs.^20^ We screen over 5800 MOFs in the publicly available CoRE MOF 2025 database to find promising structures for the separation of methane/nitrogen.^3^ The purification of methane is essential to the chemical industry and an effective measure to mitigate methane emissions into the atmosphere.^32,33^ Although industrial-scale methane separation is established within natural gas production, it often relies on energy-intensive cryogenic distillation.^34^ Recent developments focus on kinetic separation approaches and on equilibrium-based pressure-swing adsorption (PSA).^35^ Such processes using titanosilicates as adsorbents or molecular sieves are commercialized.^36^ These processes selectively adsorb nitrogen while purifying methane in the light product stream. This work aims to explore the potential of temperature-swing adsorption (TSA) processes for separating methane as the heavy product, which can be particularly interesting for feed streams with low methane concentration.^37^ Capturing methane from dilute sources remains infeasible with current materials and processes. While capturing methane from dilute sources is challenging, its global warming potential over 20 years is ∼80 times higher compared to CO_2_, offering significant decarbonization potential. This study aims to explore the feasibility of methane capture from dilute conditions (*i.e.*, coal-mine ventilation air, low-grade landfill gas, or biogas tail streams) using adsorption-based processes with MOFs. Our screening workflow allows us to analyse trade-offs between process KPI's and identify structures that perform well under diverse feed streams. For the most promising MOFs, we identify optimal process conditions to exploit the full potential of the best MOFs.

## Methods

### Grand-canonical Monte Carlo simulations

We use the RASPA 2.0 software to run the GCMC simulations.^38^ For the binary methane/nitrogen simulations, we run 20 000 initialization cycles and 100 000 production cycles to ensure that the system is equilibrated. The number of cycles is twice the number that is used in examples given by RASPA, and we evaluate the energy drift to check for equilibration. Atoms of the MOFs are described as Lennard-Jones sites using parameters from the universal forcefield (UFF),^39^ and the adsorbents are described by the TraPPE-UA force field.^40^ The cutoff distance for the Lennard-Jones interactions is 14 Å, as the TraPPE-UA force field is parametrized using this distance. We include truncated tail corrections and the Lorentz–Berthelot mixing rules. Pore blocking is evaluated using Zeo++ with 100 Monte Carlo samples per unit cell.^41^ The pore blocking radii are 1.492 Å for methane and 1.324 Å for nitrogen.^42^ This radius corresponds to 80% of the largest sphere of the TraPPE force field. The pure-component GCMC isotherms are taken from the PrISMa database, which uses similar simulation parameters.^11,42^

### Classical density functional theory simulations

We use the GPU-accelerated version of 3D cDFT.^20^ All calculations are performed on a NVIDIA GeForce RTX 4090 GPU. Similar to the GCMC simulations, the UFF force field representation describes the structure^39^ and Lorentz–Berthelot mixing rules define the solid–fluid interactions. For the fluid representation, we adopt PC-SAFT parameters^43^ and set the Lennard-Jones cutoff to 14 Å. We use a Helmholtz energy functional derived from the PC-SAFT equation of state.^44^ For the external potential, we evaluate the minimum number of grid points in each direction to obtain a grid density of 2 points per Å. This grid density was shown to give accurate results.^19^ We increase the number of grid points from this minimum number to the next power of two efficient evaluation of fast Fourier transforms. Additionally, we include pore blocking for inaccessible pores, as described for the GCMC simulations. To improve computational speed, we do not include Coulombic interactions for the studied methane/nitrogen mixture. For minimizing the grand potential in cDFT, we run 50 initial Picard iterations with a damping coefficient of 0.01, followed by a maximum of 1000 Picard iterations with a damping coefficient of 0.03. If the density profile does not converge within 1000 iterations, we run another 10 000 logarithmic Picard iterations. The convergence tolerance is set to 1 × 10^−7^, which is adjusted to lower tolerances if the calculated loading is within three orders of magnitude of the defined tolerance.

### Ideal adsorbed solution theory

In this work, we use IAST to calculate mixture adsorption. While other analytical models, such as the extended dual-site Langmuir model, have been used to extrapolate to mixtures,^16^ IAST is numerically more robust and accurate.^17^ To run the IAST calculations, we use the python package pyGaps.^45^ For each pure-component isotherm, we choose the best fit between the Langmuir, dual-site Langmuir, and triple-site Langmuir isotherm. We run IAST using the fugacity instead of the pressure to capture non-ideal behaviour at high pressures. For the GCMC isotherms, we use the Peng–Robinson equation of state, as it is used by RASPA 2.0. For the cDFT isotherms, we use the PC-SAFT equation of state.

### Clausius–Clapeyron temperature extrapolation

Temperature-swing adsorption process simulations evaluate the adsorption properties at different temperatures. A common method is the temperature extrapolation from a given adsorption isotherm at a reference temperature *T*_ref_ to a new temperature *T*_new_. For temperature extrapolation of the isotherms, we use the Clausius–Clapeyron relation to extrapolate each pressure point individually from *T*_ref_ to *T*_new_ based on the Clausius–Clapeyron equation:
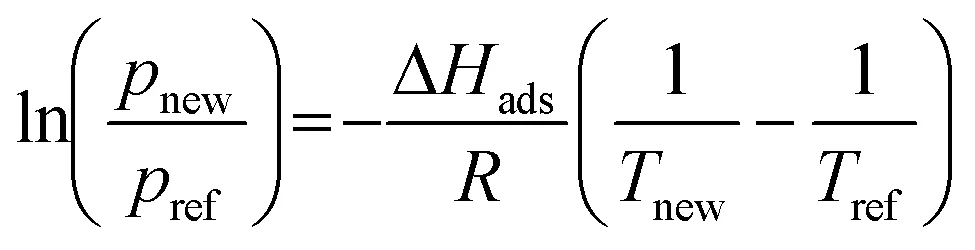
where *p*_ref_, *p*_new_, Δ*H*_ads_, and *R* denote the reference pressure, extrapolated pressure, enthalpy of adsorption and ideal gas constant, respectively. The cDFT or GCMC simulations provide the enthalpy of adsorption at each pressure.

### Temperature-swing adsorption process simulation

To describe the process, we implement an equilibrium temperature-swing adsorption model, proposed for screenings from the literature.^46^ The shortcut model implements a three-step cycle. The cycle steps are 1) adsorption, 2) heating/desorption, and 3) open cooling. The model further includes the following assumptions:

• A well-mixed bed during heating and cooling (*i.e.*, no spatial temperature or concentration gradient)

• A discontinuous step profile during the adsorption step that propagates through the column until breakthrough

• No pressure drop in the column

• Thermal and chemical equilibrium between the adsorbed and gas phase

• Heat transfer resistances are negligible, and not heat capacity of the wall is considered

Further implementation details of the model are provided in Section S1. We adapt the model in the following ways: first, instead of the ideal gas assumption, the state of the gas phase is evaluated based on the equation of state that is used for the adsorption calculation (*i.e.*, Peng–Robinson for GCMC and PC-SAFT for cDFT). Second, the enthalpy of adsorption is not evaluated from the adsorption isotherm but calculated directly from the adsorption simulations. For the validation and screening, we simulate a temperature swing from 298.15 K to 348.15 K. For the heating and cooling steps, we choose a temperature discretization of 1 K, as proposed in the original paper.^46^ The process is simulated at 1 bar. The heat capacity of the structure is estimated using the model published by Moosavi *et al.*^47^ We assume a bed void fraction of 0.37 inside the adsorption column.^48^

The process performance is evaluated by the four key performance indicators (KPI's) purity *P*, recovery *R*, working capacity WC, and thermal energy *E*, as proposed by Ajenifuja *et al.*^46^ High purity indicates a high fraction of methane in the product stream. The recovery determines, how much of the methane in the feed stream is captured in the product. Working capacity describes, how much methane a MOF can capture per cycle. Finally, the thermal energy demand is the amount of heat required to separate the methane. The KPI's are calculated as follows:1
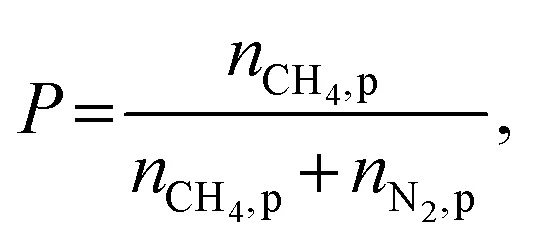
2
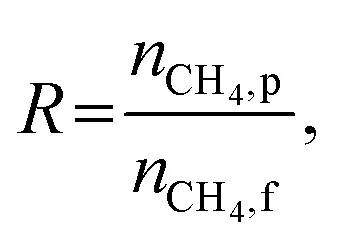
3
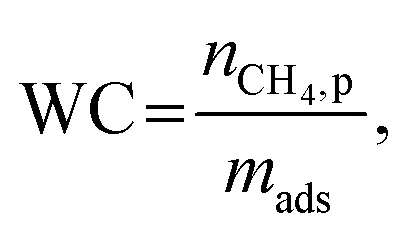
4
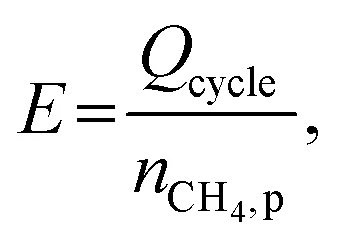
where *n*_CH_4_,p_, *n*_N_2_,p_, *n*_CH_4_,f_, *m*_ads_, and *Q*_cycle_ are the molar amount of methane in the product, the molar amount of nitrogen in the product, the molar amount of methane in the feed stream, the adsorbent mass, and the thermal energy demand, respectively. All amounts are calculated for one full temperature-swing adsorption cycle.

We recognize that the process model employed in this work is a shortcut model and that the results might change for a detailed process model. However, a detailed process model requires many further input parameters that are highly uncertain or require assumptions that are often unknown during materials screenings.^49^ Although the shortcut model has been validated against detailed models,^46,48,50^ the uncertainty of the input parameters could still affect the simulations results. In this work, we consider a shortcut model as it balances process accuracy with computational speed, which is critical for large-scale screenings.

### Validation of classical density functional theory on process metrics

The validation of cDFT aims to investigate the accuracy of cDFT predictions for process metrics. For this purpose, we first investigate how accurate cDFT reproduces binary methane/nitrogen adsorption isotherms compared to GCMC simulations. In the following, we refer to cDFT and GCMC simulations with a binary methane/nitrogen mixture as binary cDFT simulations and binary GCMC simulations. We investigate the following cDFT-based approaches:

• The first approach is the direct evaluation of mixture adsorption properties using binary cDFT simulations.

• The second approach uses cDFT to calculate full pure-component isotherms at the reference temperature *T*_ref_ = 298.15 K. The Clausius–Clapeyron equation (CC) is used to extrapolate the pure-component isotherms to other temperatures. After temperature extrapolation, the ideal adsorbed solution theory (IAST) is used to evaluate mixture adsorption from the pure-component properties. We call this approach “cDFT + CC + IAST”.

Commonly used adsorption predictions in process simulations are predicted using GCMC simulations. We compare the previously introduced cDFT-based approaches to the following GCMC-based approach:

• This third approach is based on pure-component GCMC isotherms at the reference temperature *T*_ref_ = 298.15 K. Following the same procedure as in the second approach, CC is used for temperature extrapolation and IAST for the evaluation of mixture adsorption. We call this approach “GCMC + CC + IAST”.

The PrISMa database provides pure-component GCMC isotherms for methane and nitrogen.^11,42^ From this database, we select a set of 39 MOFs that capture a diverse range of isotherm behaviours. Specifically, we aim to study MOFs spanning a wide range of Henry coefficients and saturation loadings. Fig. S1 shows the Henry coefficients and saturation loadings of all structures in the database. The choice of structures is further detailed in Section S2 in the SI.

For the 39 structures, we perform binary GCMC simulations at two temperatures and five pressure points. The choice for the pressure points is described in Section S3 in the SI. The temperatures are set to the minimum and maximum temperature of the process simulation, 298.15 K and 348.15 K. The lower temperature, 298.15 K, is the reference temperature at which all pure-component isotherms are simulated in this work and the PrISMa database.^11^ The validation at two temperatures allows us to differentiate the effects of mixture prediction at a given temperature with IAST and temperature extrapolation with the Clausius–Clapeyron equation.

In addition to the isotherm validation, we conduct process simulations for all 897 structures in the PrISMa database.^11^ For these structures, we benchmark process simulations based on cDFT + CC + IAST and binary cDFT against the state-of-the-art, GCMC + CC + IAST.

### Process-level screening of CoRE MOF 2025

After validating cDFT at the process level, we leverage its computational speed to perform a large-scale screening of MOFs for methane capture in different case studies. We screen the CoRE MOF 2025 database, which is the most recent curated database of experimentally validated MOFs.^3^ The publicly available part of the database currently contains around 5800 MOFs.

For every MOF, we simulate full pure-component methane and nitrogen isotherms with cDFT, from the Henry-regime to saturation pressure. Every isotherm contains on average 44 pressure points, leading to ∼460 000 adsorption calculations. The determination of pressure points for the full isotherm is described in Section S4 in the SI. We exclude the MOFs from our screening where one of the isotherms did not converge. For all remaining 4666 MOFs, the TSA process is simulated using the cDFT + CC + IAST approach for mixture adsorption calculations. Each MOF is evaluated based on the four process KPI's methane purity (1), methane recovery (2), working capacity (3), and thermal energy demand (4).

We perform process simulations for five methane mole fractions: 0.5, 0.1, 0.05, 0.01, and 0.003. These concentrations capture a wide range of applications from methane purification of industrial streams to biogas tail streams with the goal of finding materials that are suitable for many applications.^32,33^

For the most promising MOFs, we perform a sensitivity analysis over a range of process conditions to explore the full potential of these MOFs. The simulated process parameter ranges are as follows:

• Desorption temperature: 348.15 K to 398.15 K (in 5 K steps)

• Adsorption temperature: 288.15 K to 343.15 K (in 5 K steps)

• Pressure: 0.6 bar to 1.4 bar (in 0.2 bar steps)

We simulate all combinations of process parameters to analyse changes of the process KPI's with the operating conditions.

## Results and discussion

The results are presented in three steps: first, we validate cDFT for methane/nitrogen separation in process simulations. Second, we exploit GPU-accelerated cDFT to run a process-level screening of MOFs for multiple case studies. Finally, we perform a sensitivity analysis to find optimal process operating conditions.

### Validation of classical density functional theory

Previous studies have validated cDFT for mixtures.^31,51^ Still, these validations are limited to a few MOFs. Validating cDFT for a large number of structures allows us to understand more thoroughly the range of deviations that can appear between cDFT and GCMC simulations for mixtures. Further, the impact of deviations between cDFT and GCMC in isotherms and in the heat of adsorption on process performance has not been investigated.

**Fig. 1:**
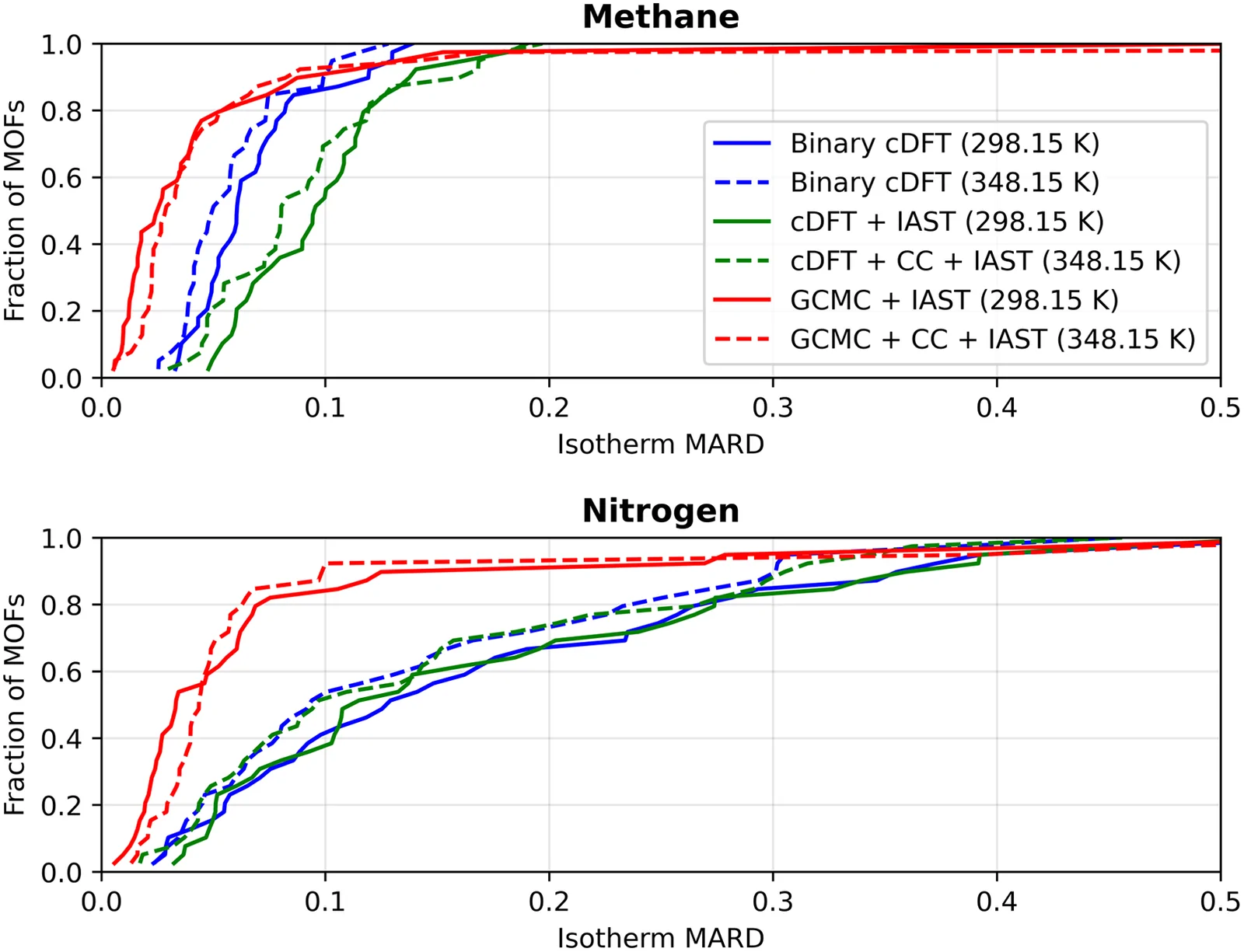
Deviations of binary isotherm predictions to binary Grand-Canonical Monte Carlo (GCMC) simulations. We plot a cumulative curve of the mean absolute relative deviation (MARD) for methane (top) and nitrogen (bottom) between each approach and binary GCMC. In total, we compare 78 isotherms (39 MOFs at two temperatures). In blue: binary cDFT. In green: pure-component cDFT at 298.15 K + Clausius–Clapeyron (CC) extrapolation to 348.15 K + ideal adsorbed solution theory (IAST). In red: pure-component GCMC at 298.15 K + CC to 348.15 K + IAST.

Overall, we observe good correspondence in the accuracy of the isotherms predicted by the three approaches, binary cDFT, cDFT + CC + IAST, and GCMC + CC + IAST, with binary GCMC simulations as the benchmark (Fig. 1). Still, deviations also occur. Since we compare the isotherms at 298.15 K and 348.15 K, we can decouple the deviation from IAST only (298.15 K) and the additional CC temperature extrapolation (348.15 K). No significant difference between the two temperatures is visible. Consequently, the CC extrapolation works well for a methane/nitrogen mixture in the temperature range between 298.15 K and 348.15 K.

For methane, both binary cDFT and cDFT + CC + IAST show very good agreement with binary GCMC. The agreement is still reasonable for nitrogen. Methane isotherms deviate by less than 10% (MARD ≤ 0.1) for 80% of the MOFs using binary cDFT and still for 50% using cDFT + CC + IAST. While the nitrogen isotherms show higher deviations for binary cDFT and cDFT + CC + IAST, still between 60% and 80% of the structures deviate below 0.2 MARD. GCMC + CC + IAST generally has lower deviations, with more than 80% of the isotherms below 0.1 MARD for methane and nitrogen. The larger deviation of cDFT for nitrogen can be explained by the lower absolute adsorption and its small quadrupole moment, which is not captured in the cDFT simulations in this work. This effect could be captured at the expense of additional calculation time.^19^

**Fig. 2:**
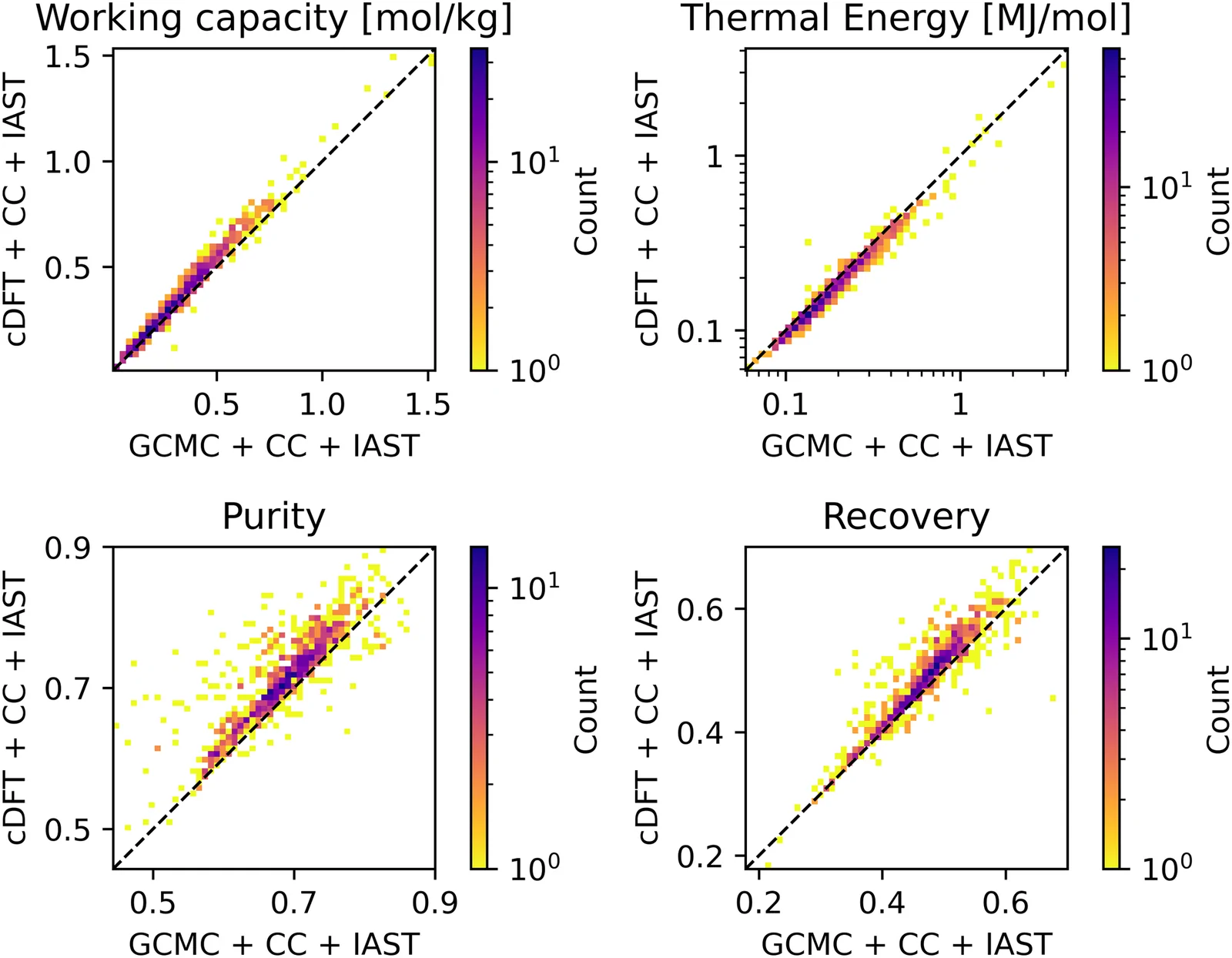
Parity plot for four process KPI's across all 819 structures. Classical density functional theory (cDFT) is compared to Grand-Canonical Monte Carlo simulations (GCMC) as reference. The point density is plotted as colour, going from purple (many structures) to yellow (few structures). The process simulations are based on pure-component isotherms from cDFT and GCMC with Clausius–Clapeyron temperature extrapolation and ideal adsorbed solution theory.

While the adsorption properties predicted by the cDFT approaches deviate more from binary GCMC than GCMC + CC + IAST, the propagation of these deviations on the accuracy of process metrics needs to be investigated to see how well cDFT is suited for process-level screenings. Thus, going beyond isotherm validation, we compare the deviation between process simulations across all structures in the database for which pure-component GCMC isotherms are available. Process simulations based on cDFT + CC + IAST are compared to GCMC + CC + IAST as the reference. Fig. 2 shows parity plots between cDFT + CC + IAST and GCMC + CC + IAST for all process KPI's.

We observe good agreement between cDFT + CC + IAST and GCMC + CC + IAST. This agreement is quantitatively confirmed by a Pearson correlation coefficient: for thermal energy and working capacity the correlation is excellent with values higher than 0.98. The correlation is somewhat lower for recovery (0.92), and purity (0.82). These findings suggest that cDFT is well-suited to screen MOFs in particular based on process-level criteria such as thermal energy and working capacity.

The cDFT approach shows a small bias towards higher purity, recovery, and working capacity and towards lower thermal energy demand. The reason is a slight overprediction of methane adsorption by cDFT compared with GCMC (Fig. S4). Additionally, the isotherms from cDFT + CC + IAST deviate from GCMC + CC + IAST about the same as GCMC + CC + IAST from binary GCMC. Consequently, we also expect these models to deviate similarly at the process-level.

**Fig. 3:**
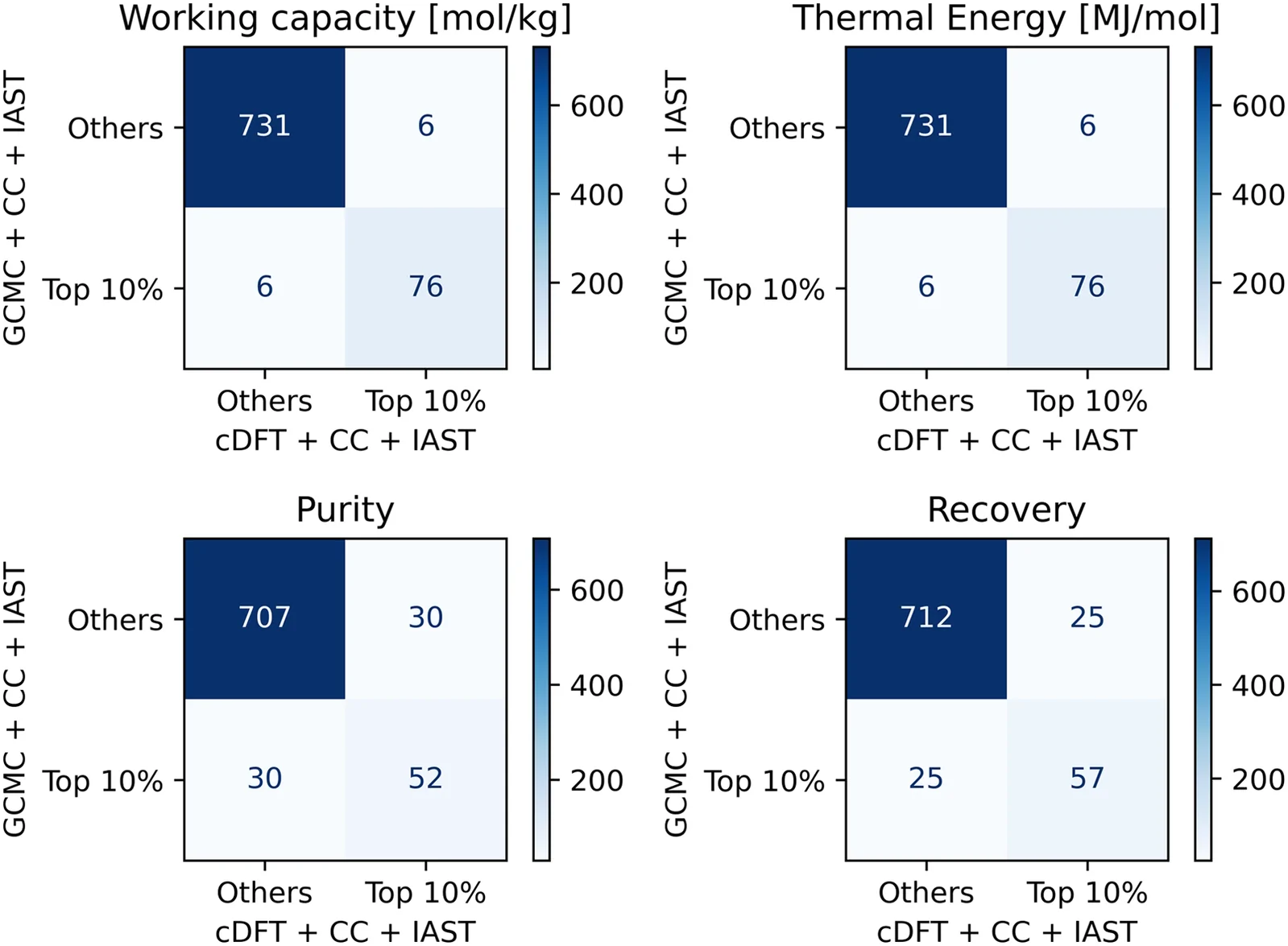
Confusion matrix between the process simulations based on cDFT + CC + IAST and GCMC + CC + IAST. We categorize the structures based on their ranking for each process KPI into structures that are among the best 10% of the KPI and others. Then, we compare these categories between cDFT + CC + IAST and GCMC + CC + IAST.

Ultimately, a screening approach aims to find the best MOFs. Since the MOF ranking is the main interest for a screening, we compare which structures are identified among the best 10% for each process KPI (Fig. 3). cDFT correctly identifies more than 92% of the best MOFs for thermal energy demand and working capacity showing excellent agreement with GCMC on the process level. For purity and recovery, cDFT still captures between 60% and 70% of the best MOFs. We choose the ten percent threshold to balance the trade-off between the accuracy of the purity and recovery predictions and the number of best materials (Fig. S3). Regarding the energy demand and working capacity, the best MOFs are identified reliably, independent of the threshold.

For many applications, purity and recovery must reach a minimum threshold, but energy demand or working capacity are the KPIs that are optimized, since they relate to CAPEX (working capacity) and OPEX (energy). Consequently, accurate predictions for the energy demand and working capacity confirm cDFT as a useful screening tool.

Additionally, we run all process simulations with binary cDFT. In this case, cDFT simulations are used for every adsorption calculation of the process simulation. We compare both cDFT + CC + IAST and binary cDFT with GCMC + CC + IAST as reference (Fig. S2). Binary cDFT is one percent more accurate in terms of mean absolute relative error compared to cDFT + CC + IAST (Table S1). However, one process simulation with binary cDFT takes around 30 minutes, while cDFT + CC + IAST requires only 1–2 minutes. Consequently, we regard cDFT + CC + IAST as the better trade-off between accuracy and computational cost. For mixtures that cannot be predicted well with IAST, it could be beneficial to run process simulations with binary cDFT for higher accuracy, as binary cDFT is still faster than GCMC + CC + IAST.

In addition, we correlate the isotherm deviation with the process deviation. As expected, larger deviations between cDFT and GCMC in isotherms results in larger deviations in process KPI's (Fig. S4). Structures with an isotherm mean relative deviation below 20% are accurately predicted in process simulations (*e.g.* with a purity deviation below 5%).

Generally, the process-level validation supports the sufficient accuracy of cDFT for screening. In particular, the computational speedup of cDFT compared to GCMC justifies the choice of cDFT for large-scale process-level screenings. The best screening materials from cDFT can be afterward validated and further analysed with GCMC and detailed process simulations; however, this analysis lies outside the scope of this work.

### Screening of CoRE MOF 2025

To demonstrate the power of cDFT-based screenings, we screen all publicly available structures of the CoRE MOF 2025 database.^3^ The CoRE MOF database is the latest published database of experimentally validated MOFs. The publication of CoRE MOF 2025 includes a recommended screening list for structures. All structures in this database were curated and should represent valid MOFs that have been synthesized.

**Fig. 4:**
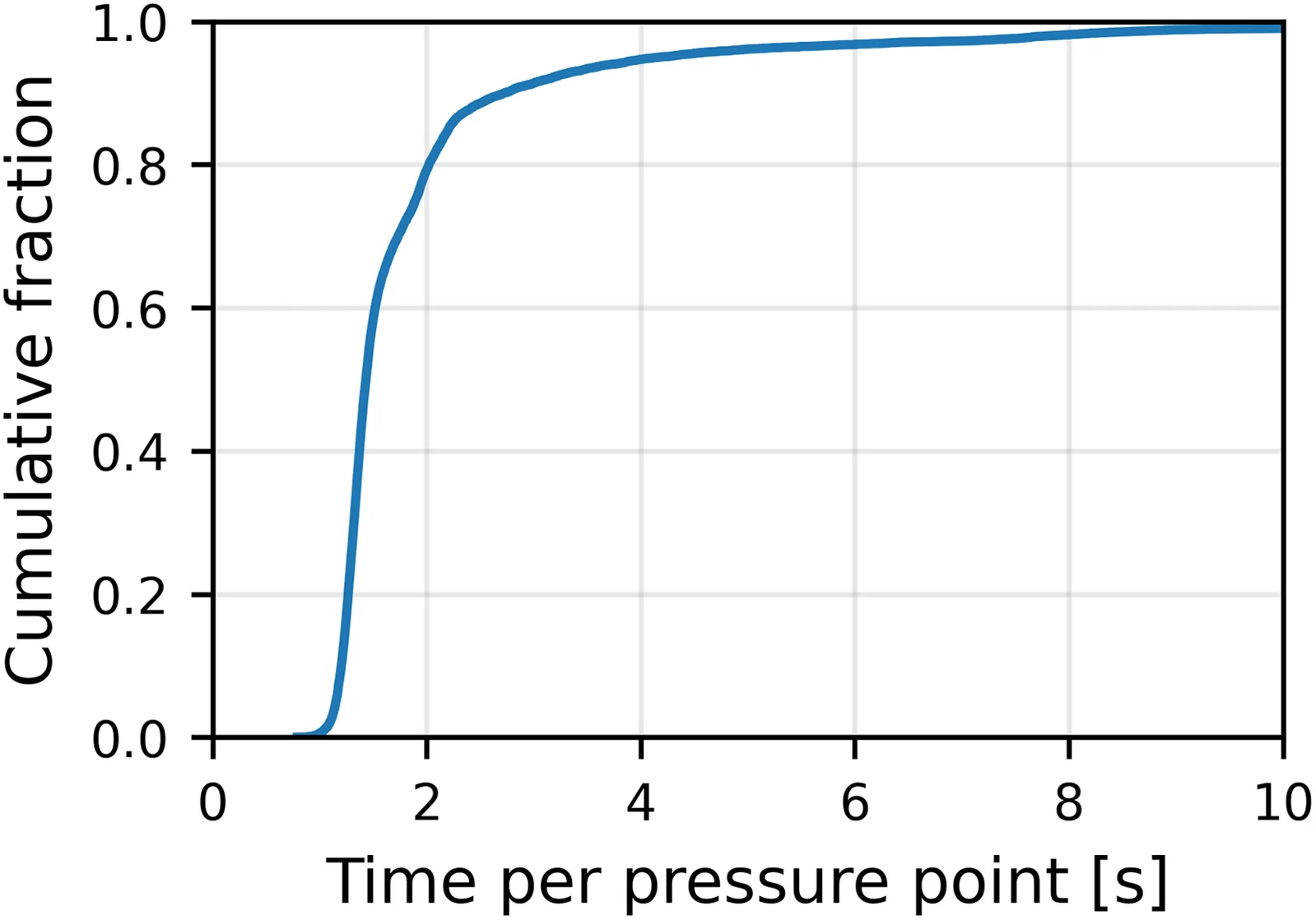
Cumulative curve of the cDFT calculation time per pressure points for all 4666 MOF structures in CoRE MOF 2025. The cDFT calculations yield the adsorption loading and the enthalpy of adsorption. The slowest one percent of structures lies between ten and 59 seconds.

First, we simulate full pure-component isotherms for methane and nitrogen. Out of ∼5800 structures, 5155 methane isotherms, 5238 nitrogen isotherms converged, and 4666 structures converge for both. In total, we evaluate over 460 000 pressure points, with an average time per pressure point of 1.9 s (Fig. 4).

The average evaluation time per isotherm was 84 s. The entire screening took only 5 days, running on 2 GPUs in parallel. The calculation time depends on the size of the structure: large structures contain more grid points, leading to longer computation times. The effect of the unit cell size becomes visible for structures that are larger than ∼(23 Å)^3^, corresponding to ∼130 000 cDFT grid points. We show the calculation time per pressure point as a function of the number of cDFT grid points in Fig. S5.

For every structure, we run a TSA process simulation using the cDFT + CC + IAST approach for an equimolar mixture of methane/nitrogen. For each MOF, we evaluate four process KPI's. Ideally, MOFs should perform well for the four KPI's as, for example, a MOF that reaches low purity would not be promising even if it requires less energy.

**Fig. 5:**
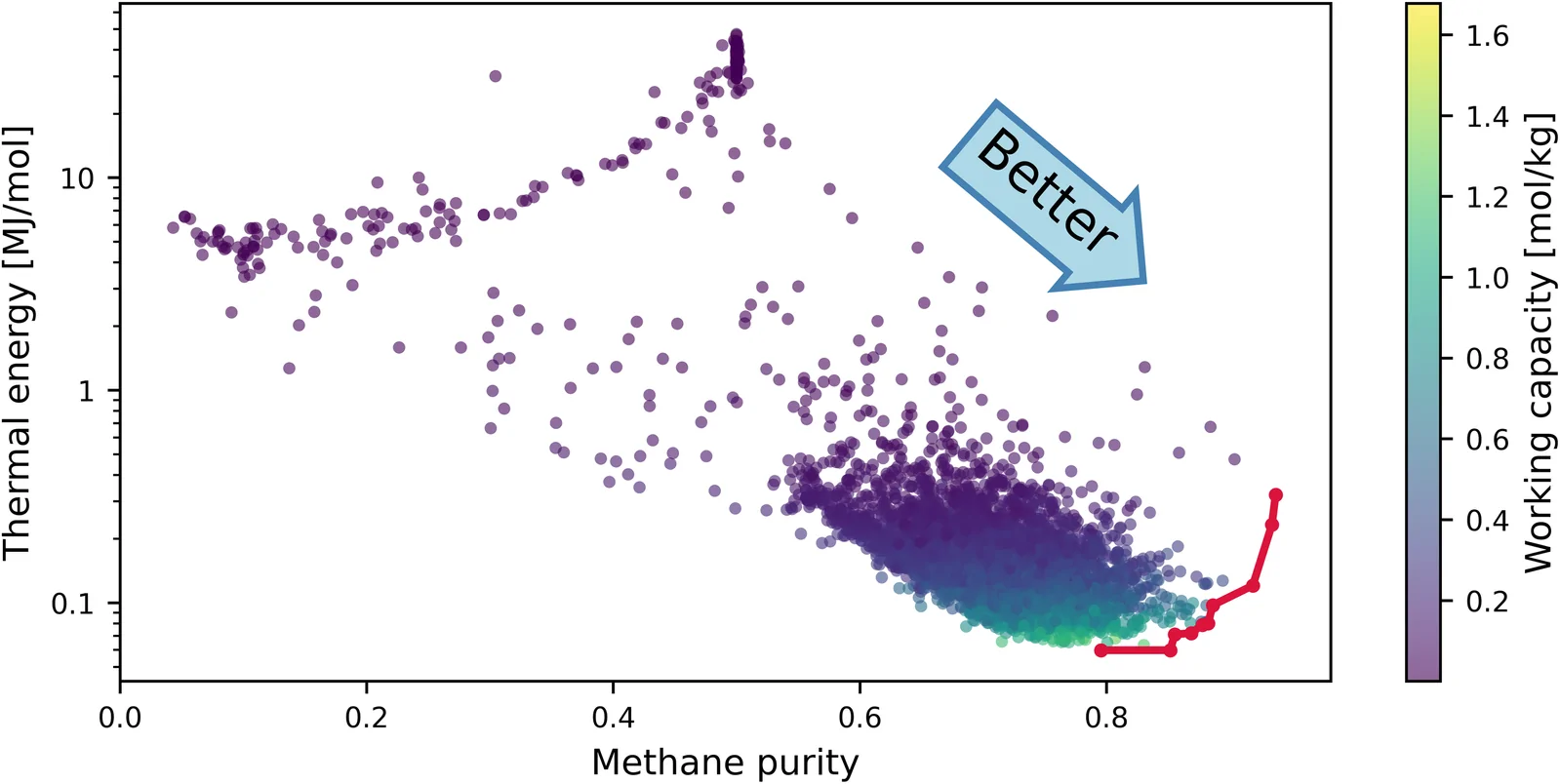
Process screening of 4666 MOFs. For each MOF, we plot the methane purity (*x*-axis) against the thermal energy demand (*y*-axis) and indicate the working capacity as colour. We consider an equimolar methane/nitrogen feed mixture. We mark the Pareto front of the best MOFs as red line.

Fig. 5 shows the trade-off between methane purity and thermal energy demand. Many structures lie in the corner of high purity and low energy demand. However, a Pareto front between the MOFs with the highest purity and the lowest energy demand shows a trade-off between the KPI's: while the MOF with the lowest energy demand of 0.06 MJ mol^−1^ reaches a purity of 80%, the MOF with the highest purity of 94% requires 5 times more energy (0.32 MJ mol^−1^). Thermal energy demand and working capacity are well-correlated for an equimolar feed: when more methane per cycle is captured (higher working capacity), the thermal energy demand per captured mole of methane decreases (Fig. S6). Noteworthy, purity and recovery are also well-correlated for an equimolar feed (Fig. S7). The structures with a high purity also achieved high recovery (*cf.* Fig. S7), which is particularly relevant for the separation of industrial streams with high methane concentrations.

### Screening for different feed compositions

In the previous section, we analyse a process for a fixed methane feed concentration of *x*_CH_4_,feed_ = 0.5. Next, we extend the process analysis to different feed compositions to evaluate the impact of the methane feed concentration on the MOF performance. The goal is to identify materials that perform well at different feed concentrations to enable multiple applications – from purification of methane point sources to methane capture at dilute conditions. This analysis requires process simulations at different feed compositions. For comparing different feed compositions, we define the enrichment as
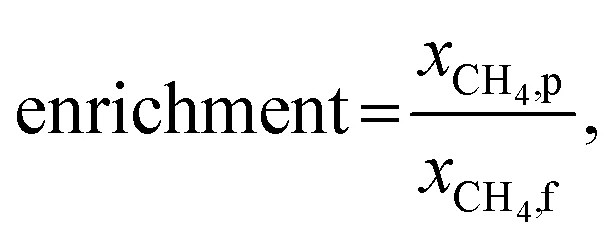
where *x*_CH_4_,p_ and *x*_CH_4_,f_ represent the mole fraction of methane in the product and the feed stream, respectively. The absolute purity decreases significantly for dilute conditions. Therefore, enrichment allows for better comparison.

**Fig. 6:**
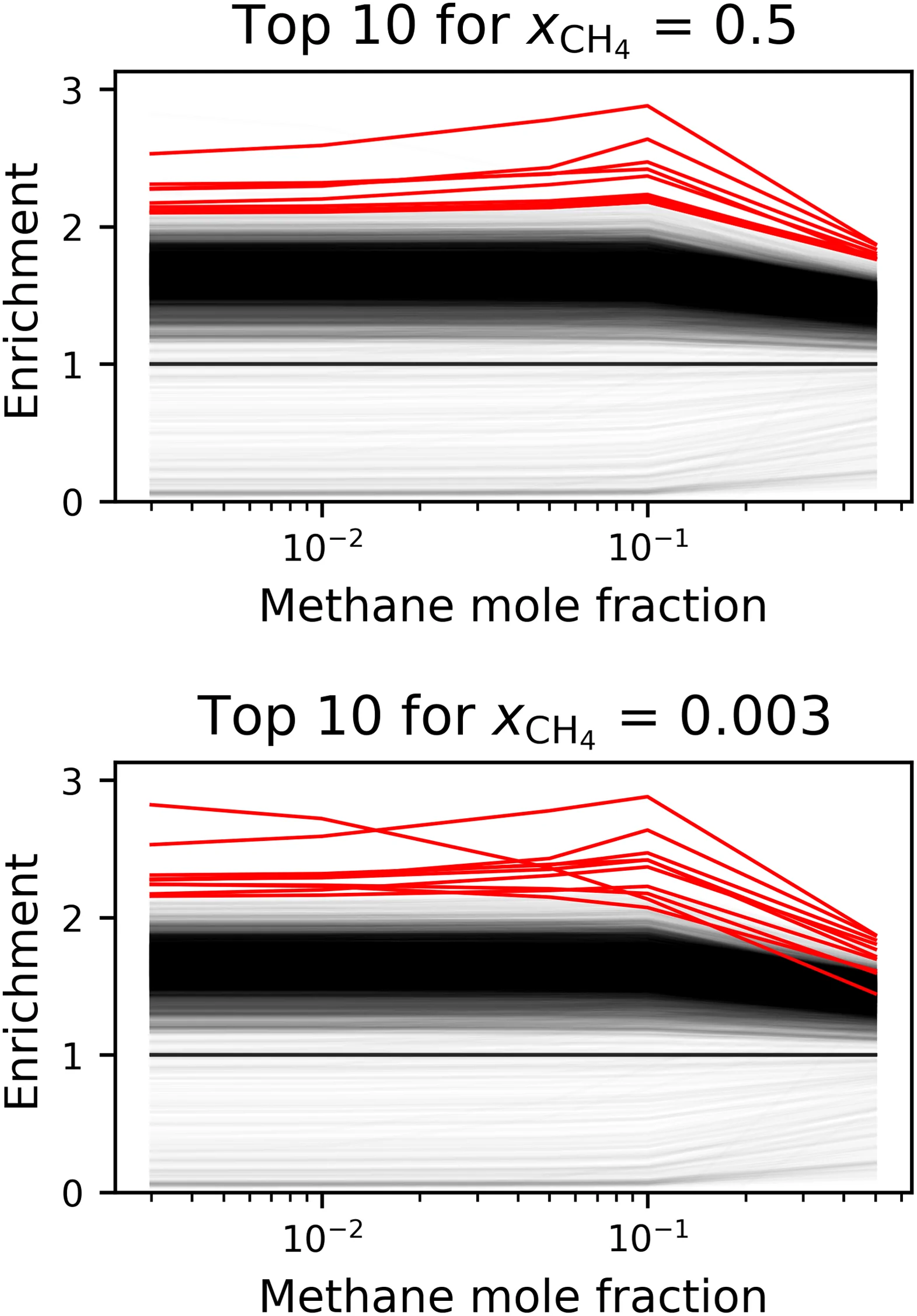
Methane enrichment across different feed compositions. We show the enrichment of methane, defined as *x*_CH_4_,product_/*x*_CH_4_,feed_, for all MOFs simulated at five methane concentrations in the feed stream: 0.5, 0.1, 0.05, 0.01, and 0.003. We highlight the best ten structures for the highest (top figure) and lowest (bottom figure) methane feed concentration in red.

Many MOFs show a constant enrichment across different feed compositions (Fig. 6). The best 10 MOFs for high methane concentrations are also among the 22 best MOFs at dilute conditions. However, among the best MOFs for low feed concentrations, three MOFs show significant performance loss at higher feed concentrations. These MOFs are in the top 10 for a methane feed concentration of 0.003, but rank 180th, 254th, and 2187th for a methane feed concentration of 0.5. In Fig. S9, we show the performance across different feed concentrations for the other process KPI's.

MOFs that are strongly selective for methane may not fully regenerate for a desorption temperature of 348.15 K at a lower methane feed concentration (Section S9). Increasing the desorption temperature improves the regeneration of these MOFs and thereby also the recovery. This result demonstrates that the case study (*i.e.*, the feed stream to separate and the temperature available for desorption) can affect the performance of each MOF differently, motivating the use of an application-specific analysis on the process level. We conducted additional simulations for all feed concentrations with a desorption temperature of 448.15 K (Fig. S12 and S13), showing that a higher desorption temperature increases the regeneration and thereby the performance of strongly selective MOFs. For low feed concentrations, we observe a constant enrichment, independent of the feed concentration. As the enrichment, along with the other process KPI's, is strongly dependent on the process conditions and the process cycle, it could be further improved by modifying the conditions or the cycle itself, *e.g.*, by adding a vacuum step. Our framework enables this integration and extension of the process for further development.

Along with a low recovery for a desorption temperature of 348.15 K, the working capacity is in the order of 10^−2^ mol kg^−1^ for the lowest feed concentration of *x*_CH_4_,feed_ = 0.003. Consequently, a lot of nitrogen is cycled. To achieve more technically feasible performance for dilute methane concentrations, higher desorption temperatures (Section S10) and cycle adaptations are necessary and can be extended in future work.

Although the MOFs do not fully recover at low feed concentrations, we aim to find overall promising MOFs for the given desorption temperature range. A ranking based on enrichment allows to identify structures with high potential. We choose the best materials for dilute and concentrated methane feeds based on the following conditions:

• Among the best 750 MOFs for both purity and energy demand at the respective feed composition

• High enrichment: at every feed composition, the enrichment must be at least 90% of the best enrichment among all MOFs

• Largest free sphere >5 Å to avoid diffusion limitation, as materials with very small pores can exhibit slow mass transfer, impeding the process performance^52^

• No open metal sites, to avoid strong interactions with carbon dioxide and potentially water^53,54^

We identify five MOFs with promising performance across all feed compositions (Table 1). All structures that are analysed below are associated with PACMAN partial charges and are part of the “all-solvent-removed” (ASR) subset. In the following tables and figures, we omit the “_ASR_pacman” suffix from the original filename. For these structures, we validate the cDFT isotherm predictions with binary GCMC simulations. The validation is further detailed in Section S11 in the SI. cDFT and GCMC deviate between 0.05 and 0.15 for methane and between 0.1 and 0.2 for nitrogen, which is the expected range, shown in Fig. 1. Based on the correlation between isotherm and process deviation in Fig. S4, we estimate the expected deviation of our process simulations to be up to 5% for purity and recovery, and up to 10% for working capacity and thermal energy demand, but we do not expect higher deviation. Consequently, the identified MOFs can be promising materials for the studied applications, but implementation would most likely require higher desorption temperatures, in particular, at low methane concentrations. The presented method provides a starting point for further process optimization and design to confirm the potential of promising MOFs.

**Table 1:** MOFs with promising process performance. The four process KPI's purity, recovery, working capacity and energy demand are evaluated for a methane feed concentration of *x*_CH_4__ = 0.5. The enrichment is given for both case studies of a high and a low methane feed concentration

MOF name	Purity [—]	Recovery [—]	Working capacity [mol kg^−1^]	Energy demand [MJ^−1^ mol]	Enrichment @*x*_CH_4__ = 0.5	Enrichment @*x*_CH_4__ = 0.003	Image
UTULUR	0.75	0.51	0.48	0.103	1.50	1.82	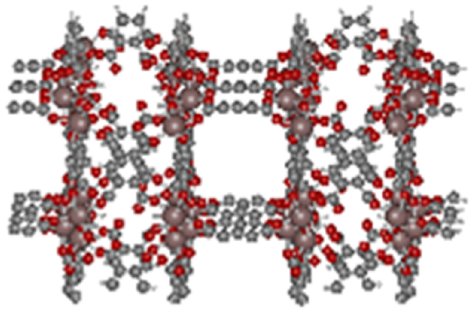
am7b17755_si_002	0.75	0.52	0.90	0.075	1.51	1.86	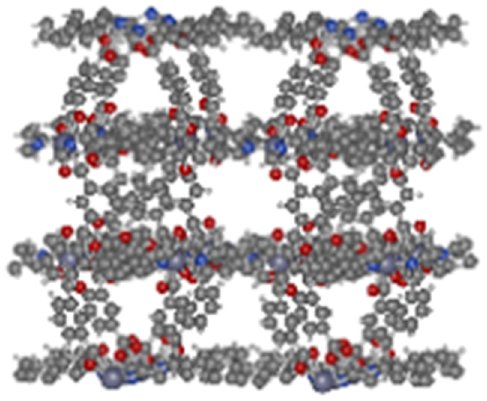
NIRPAI	0.80	0.58	1.68	0.060	1.59	1.87	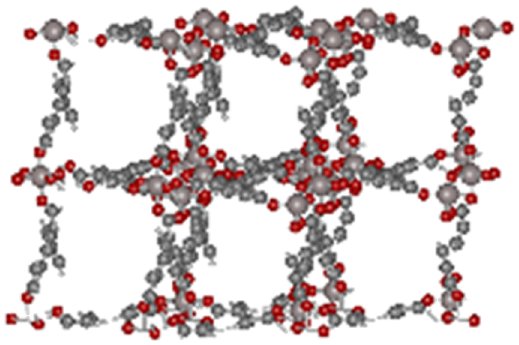
TAKTAD	0.77	0.54	1.08	0.065	1.54	1.80	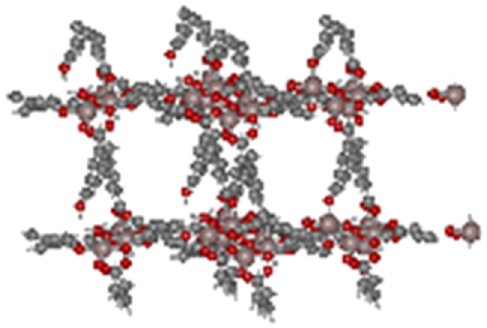
OTIPAK	0.77	0.52	0.69	0.084	1.54	1.78	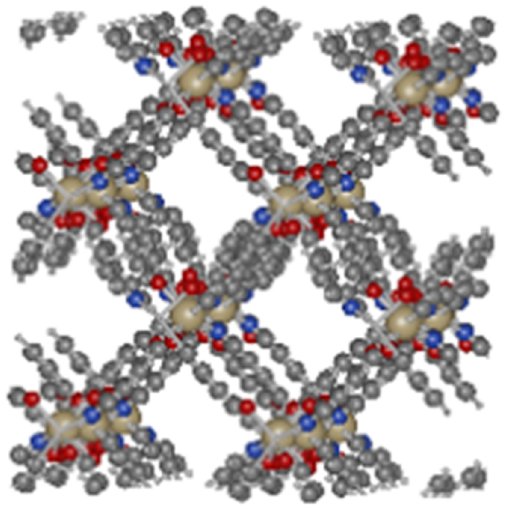

### Sensitivity analysis of process parameters

The previous analysis focuses on a fixed set of process parameters. Adapting the process parameters for each material and application can improve the process performance. We analyse trends in the process performance through sensitivity analysis of the process parameters, namely the adsorption and desorption temperatures, as well as the operating pressure. We conduct the sensitivity analysis for all five promising MOFs and observe the same trends across all MOFs. We show the simulation results of UTULUR as one representative example in Fig. S15.

The results show that all process KPI's improve with lower adsorption and higher desorption temperatures. This trend is expected for purity, recovery, and working capacity. However, for the energy demand, it is not trivial to estimate such a behaviour: while the absolute energy demand increases with a larger temperature swing, more methane is captured as well. The higher methane capture outweighs the higher energy demand. Consequently, the specific energy demand decreases. This trade-off can only be captured in process simulations and not based on the adsorption properties. For the operating pressure, no optimal pressure improves all process KPI's, but we identify a trade-off between process KPI's: while lower pressure yields higher purity and recovery, it reduces the working capacity and increases the specific energy demand. Depending on the application, the operating pressure can be a degree of freedom (*e.g.*, industrial streams) or defined by the application (*e.g.*, methane capture from low-grade landfill gas or biogas tail streams). If the operating pressure is a degree of freedom, it should be chosen based on the desired product and process performance.

**Fig. 7:**
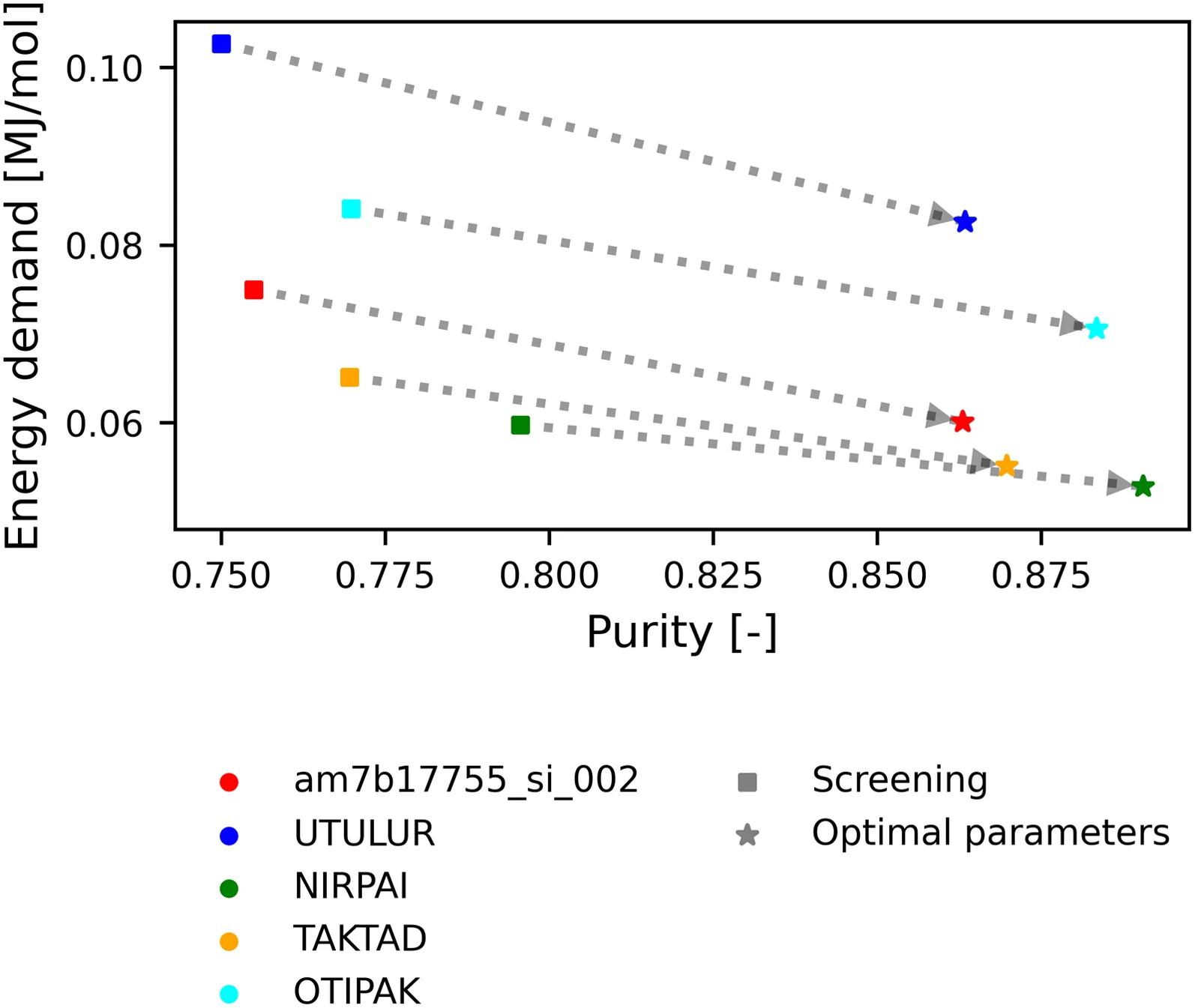
Optimal process results for promising structures. The optimal processes (stars) minimize the energy demand for the five promising structures. The results are compared to the process screening results (squares). We consider an equimolar methane/nitrogen feed mixture.

In Fig. 7, we show the process simulation with minimal energy demand of the five best structures. The minimal energy demand is achieved for *T*_ads_ = 288.15 K, *T*_des_ = 398.15 K, and *p* = 1.4 bar for all structures. The values correspond to the bounds of the sensitivity analysis. Similar trends with optimal operating conditions at the bounds were observed before for the simulation of shortcut models.^46,50^ We compare the results for the optimal conditions with the screening simulation results. All structures also improved methane purity. Noteworthy, the ranking of the structures in terms of energy demand does not change. For purity, the ranking of the best three MOFs does not change. Only UTULUR_ASR and am7b17755_si_002_ASR, which have a very similar purity, change their ranking. These findings indicate that the process simulation of the large-scale screening enables us to identify promising structures without process optimization of each structure.

## Conclusions

We successfully demonstrate that 3D classical density functional theory serves as an accurate and efficient method for predicting methane/nitrogen mixture adsorption properties. In particular, its accuracy extends beyond isotherm predictions to the process level. Process performance predictions based on cDFT inputs match simulations based on computationally expensive GCMC data well.

The primary advantage of cDFT is the exceptional computational speed which enables large-scale screenings at the process level. We screen the whole publicly available CoRE MOF 2025 database, comprising approximately 5800 structures, in only five days. This screening represents a significant acceleration in material discovery, allowing for the evaluation of full pure-component isotherms and subsequent process simulations in a fraction of time compared to state-of-the-art methods.

Our approach enables us to screen all structures over many feed compositions and process conditions. This analysis demonstrates two advantages: first, the process simulations at different feed compositions identify materials performing well for the general separation of methane/nitrogen across applications. Second, the sensitivity analysis for process conditions unveils trends to further improve process performance. Future work can consider validation of the most promising identified MOFs with experiments and detailed process models.

Ultimately, this work establishes a new screening paradigm that combines the computational speed of cDFT with process modelling to rapidly identify promising MOFs for diverse industrial applications.

## Author contributions

M. G.: conceptualization, data curation, formal analysis, investigation, methodology, project administration, software, validation, visualization, writing – original draft; V. D.-D.: conceptualization, methodology, supervision, writing – review & editing; P. R.: methodology, software, supervision, writing – review & editing, J. G.: funding acquisition, methodology, project administration, resources, writing – review & editing; A. B.: conceptualization, funding acquisition, methodology, project administration, resources, supervision, writing – review & editing.

## Conflicts of interest

The authors declare the following financial interests and personal relationships that could be considered as potential competing interests: A. B. has served on review committees for research and development at ExxonMobil and TotalEnergies, companies active in both oil and gas and chemical production. A. B. holds ownership interests in firms that provide services to industry, some of which may operate adsorption processes. The remaining other authors declare no known competing financial interests or personal relationships that could have influenced the work reported in this paper.

## Supplementary Material

ME-011-D6ME00066E-s001

## Data Availability

This study was carried out using publicly available data from the PrISMa database at https://doi.org/10.5281/zenodo.11244258 and from the CoRE MOF 2025 database at https://doi.org/10.5281/zenodo.15621349. Data for this article, including the cDFT adsorption isotherms, the enthalpies of adsorption, the process simulation results, and GCMC input files and simulation results are available at Zenodo at https://doi.org/10.5281/zenodo.19331046. Supplementary information (SI) is available. See DOI: https://doi.org/10.1039/d6me00066e.
