# Supplementary material for: Fast process-level screening of metal–organic frameworks for adsorption separation using 3D classical density functional theory
Source: Mol Syst Des Eng. 2026 Jun 27;11(11):1056–67. doi: 10.1039/d6me00066e (PMC13504625; doi:10.1039/d6me00066e)
Supplement: Supplementary file 1 [file ME-011-D6ME00066E-s001.pdf]

Supplementary Information (SI) for Molecular Systems Design & Engineering.
This journal is © The Royal Society of Chemistry 2026

Fast process-level screening of metal-organic frameworks for adsorption separation using 3D classical density functional theory

Marcel Granderath[a], Philipp Rehner[a], Joachim Gross[b], Vincent Dufour-Décieux*[a] and André Bardow*[a]

[a] Energy & Process Systems Engineering, Department of Mechanical and Process Engineering, ETH Zurich, Tannenstrasse 3, 8092 Zurich, Switzerland.
[b] Institute of Thermodynamics and Thermal Process Engineering, University of Stuttgart, Pfaffenwaldring 9, 70569 Stuttgart, Germany.
* Corresponding author, vdufour@ethz.ch, abardow@ethz.ch

S1: Temperature-swing adsorption process model

The temperature-swing adsorption model, proposed in the literature[1], is an equilibrium-based shortcut model that uses a three-step cycle. The approach starts by simulating the heating (desorption) step. The adsorption step is assumed as a discontinuous step profile, propagating through the column. Thus, the column is fully saturated after the adsorption step, and the initial state of the desorption is known. This assumption allows the model to achieve cyclic steady state within one process cycle. During desorption and cooling, the temperature swing is discretized into small steps. At each temperature step k, the model solves two mole balances, one for each component (A and B):

$$N_{A,tot}^{k-1} - y_A^k N_{out}^k - N_{A,tot}^k = 0$$

$$N_{B,tot}^{k-1} - (1 - y_A^k) N_{out}^k - N_{B,tot}^k = 0$$

where $N_{A,tot}^k$, $N_{B,tot}^k$, N_{out}^k, and y_A^k are the total moles of component A, the total moles of component B, the amount of moles leaving the column, and the mole fraction of component A in the column at temperature step k. The two equations are solved iteratively, with the two unknowns N_{out}^k and y_A^k. The total moles of one component are composed of the sum of the moles in the gas phase and the moles in the adsorbed phase. The moles in the gas phase are defined by the temperature, pressure, and volume. The equation of state used to calculate the moles in the gas phase is PC-SAFT for cDFT and Peng-Robinson for GCMC, consistent with the adsorption calculations. The moles in the adsorbed phase are calculated based on the given temperature, pressure, and gas phase composition.
After the two mole balances are solved, the energy balance is used to evaluate the thermal energy demand:

$$mc_p \Delta T = \sum_i \Delta H_i \Delta N_{i,ads} + Q_{ext}$$

where m, c_p, ΔT, ΔH_i, $\Delta N_{i,ads}$, and Q_{ext} denote the adsorbent mass, the adsorbent heat capacity, the temperature step, the enthalpy of adsorption of component i, the change of the adsorbed amount of component i, and the thermal energy demand. The enthalpy of adsorption is known from the adsorption simulations, and the heat capacity is predicted using the machine learning model of Moosavi et al.[2] In our calculations, we assume an adsorbent mass of $1\ kg$. The temperature difference is fixed, and the change in the adsorbed amount is known from the mole balances.

As we assume a discrete step-change for the adsorption, the amount adsorbed can be directly evaluated from the mole balances, given the column states before and after the adsorption step:

$$N_{A,tot}^{before} + y_{A,feed} N_{feed} - N_{A,tot}^{after} - y_A^{before} N_{waste} = 0$$

$$N_{B,tot}^{before} + (1 - y_{A,feed}) N_{feed} - N_{A,tot}^{after} - (1 - y_A^{before}) N_{waste} = 0$$

The equations are solved for the two unknowns N_{feed} and N_{waste}.

S2: Choice of structures for validation

We choose the structures from the PrISMa database to representatively cover the material space.[3] For each structure, we compute full pure-component isotherms for methane and nitrogen using classical density functional theory (cDFT).[4] We estimate the pore-filling pressure as the pressure where the loading crosses 50% of the maximum loading. This approximates the pressure where a step from low loading to saturation occurs. We choose a set of structures that represent a wide spread of pore-filling pressures and cover a wide range of maximum loading and Henry coefficient. Further, we omit structures where any simulation during the validation (cDFT or GCMC) did not converge. In Figure S1, we display the chosen structures within the space of the entire database. We show the Henry coefficients and saturation loading for all structures. The chosen structures are marked red and cover the full space of structures.

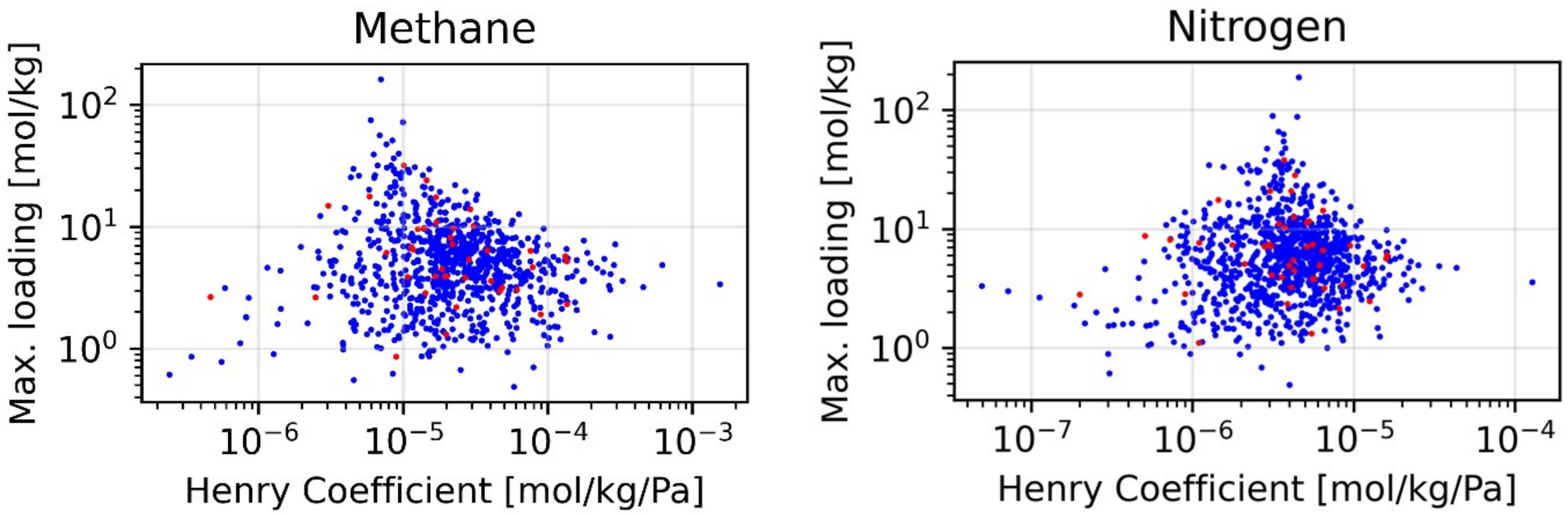

Figure S1: Choice of structures for isotherm validation. The Henry coefficient (x-Axis) against the maximum loading (y-Axis) for the methane (left) and nitrogen (right) isotherms. The red dots are the structures chosen for validation of classical density functional theory (cDFT) and they are meant to cover a wide space of MOF characteristics.

S3: Choice of pressure points for validation

To validate the binary isotherm predictions of three methods (binary cDFT, cDFT + CC + IAST, and GCMC + CC + IAST) against binary GCMC benchmarks, we aim to sample pressure points that capture varying loading levels for both components. The lowest pressure point represents a low loading for both methane and nitrogen. For each component, we determine the pressure that correspond to 10% of the maximum loading. Then, we average the two pressures to obtain the lowest pressure point. For the highest pressure, we follow the same procedure, but for 90% of the maximum loading, where both components are close to saturation.

In between, we choose three pressure points corresponding to 25%, 50%, and 75% of the maximum loading of each component to cover the region where one component has a high loading and the other component a low loading. These intermediate pressures are also averaged between the components. Therefore, each isotherm includes five pressure points in total.

S4: Determination of pressure points for full adsorption isotherms

First, we choose a low starting pressure point based on the following heuristic equation:

$$\frac{p_{start}}{Pa} = 10^5 * \exp\left(0.5177 * \min\left(\frac{ext.pot.}{K}\right) + 3.1377\right),$$

where p_{start} is the starting pressure point in Pascal and $\min\left(\frac{ext.pot.}{K}\right)$ is the minimum energy of the external potential field in Kelvin, which is calculated for cDFT. We determined the equation based on existing GCMC adsorption isotherm data. Additionally, we choose two pressure points at 50% and 25% of the starting pressure. We fit a linear model through the loading at the three pressures. If the R^2 of the linear fit lies above 0.99, the three pressures are in the Henry regime. If the R^2 is lower, we add a pressure point at 50% of the lowest pressure and repeat the procedure until we successfully reach the Henry regime.

In the next step, we determine the pressure points until saturation. Between two pressure points, we set a maximum loading change to $0.2\frac{mol}{kg}$. Based on that value, we determine the next pressure point from the slope of the isotherm from previous pressure points. Additionally, we constrain a general maximum pressure step size to 120 times the low starting pressure. We assume to reach saturation when the loading change between two pressure points is below $0.01\frac{mol}{kg}$. The resulting isotherms typically range from the order of 10 to $100\ Pa$ to the order of $1000\ bar$.

S5: Comparison of process deviation for binary cDFT

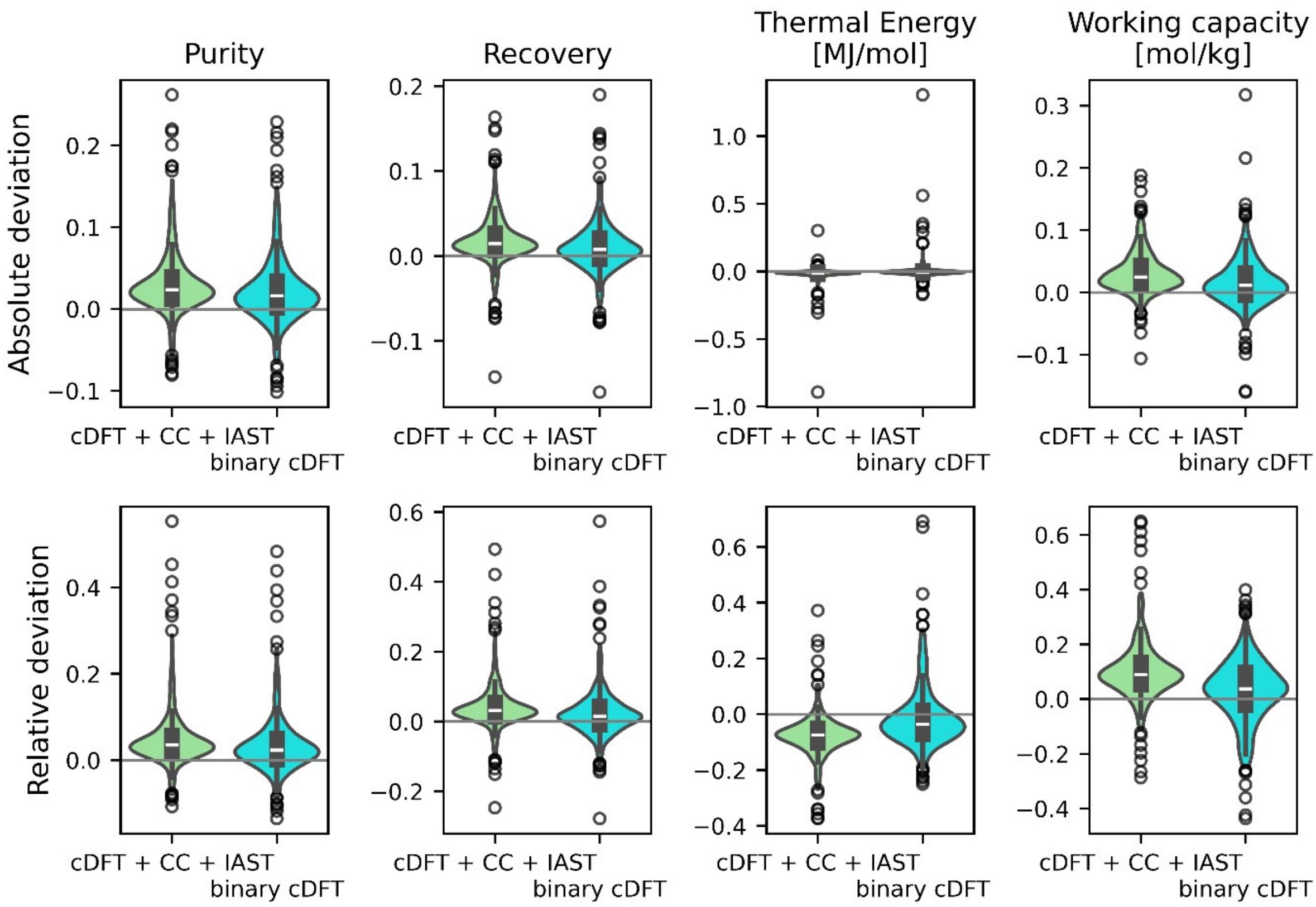

Figure S2: Process deviation for four process KPI's across 622 converged structures. We show the deviation between approaches based on classical density functional theory (cDFT) with grand-canonical Monte Carlo simulations (GCMC) as a reference. The left approach as well as the GCMC reference base on full pure-component isotherms, coupled with Clausius-Clapeyron (CC) temperature extrapolation and ideal adsorbed solution theory (IAST). For the right approach, we run binary cDFT calculations for every adsorption calculation in the process simulation. Absolute deviations are calculated as │cDFT – GCMC│, relative deviations as │(cDFT – GCMC) / GCMC |. The 1 % with the highest and lowest deviation are shown as individual circles. Since some processes with binary cDFT did not converge, the analysis of this figure contains fewer structures than Figure 2 and Figure 3 in the main text.

Table S1: Process deviations of binary cDFT and cDFT + CC + IAST. Both cDFT approaches are compared to GCMC + CC + IAST as a reference.

Property	Method	Mean absolute error	Mean absolute relative error
Purity	Binary cDFT	0.035	5.3%
	cDFT + CC + IAST	0.031	4.6%
Recovery	Binary cDFT	0.023	5.0%
	cDFT + CC + IAST	0.020	4.3%
Working capacity [mol/kg]	Binary cDFT	0.034	11%
	cDFT + CC + IAST	0.028	9.1%
Thermal energy demand [MJ/mol]	Binary cDFT	0.021	8.6%
	cDFT + CC + IAST	0.022	7.6%

S6: Comparison of isotherm and process deviation

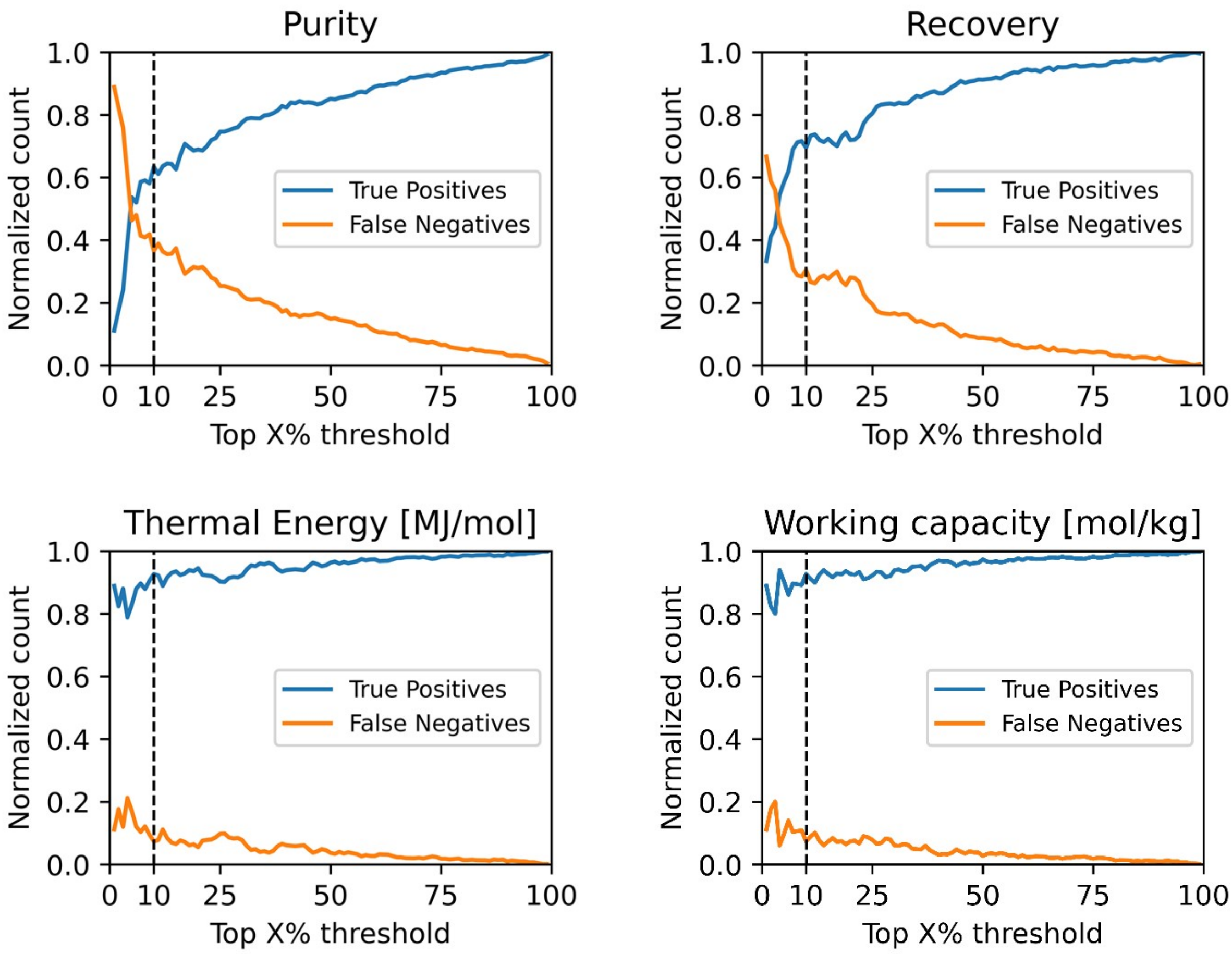

Figure S3: Classification trajectory of the best MOF, compared between cDFT + CC + IAST and GCMC + CC + IAST. For both approaches, we classify the MOFs by the best x%. Then, we correlate cDFT + CC + IAST with GCMC + CC + IAST as reference and normalize the true positives and false negatives (the bottom row of the confusion matrix in Figure 3) based on the total number of positives (the sum of both values). We indicate the 10% threshold, described in the main text, as a dashed-black line.

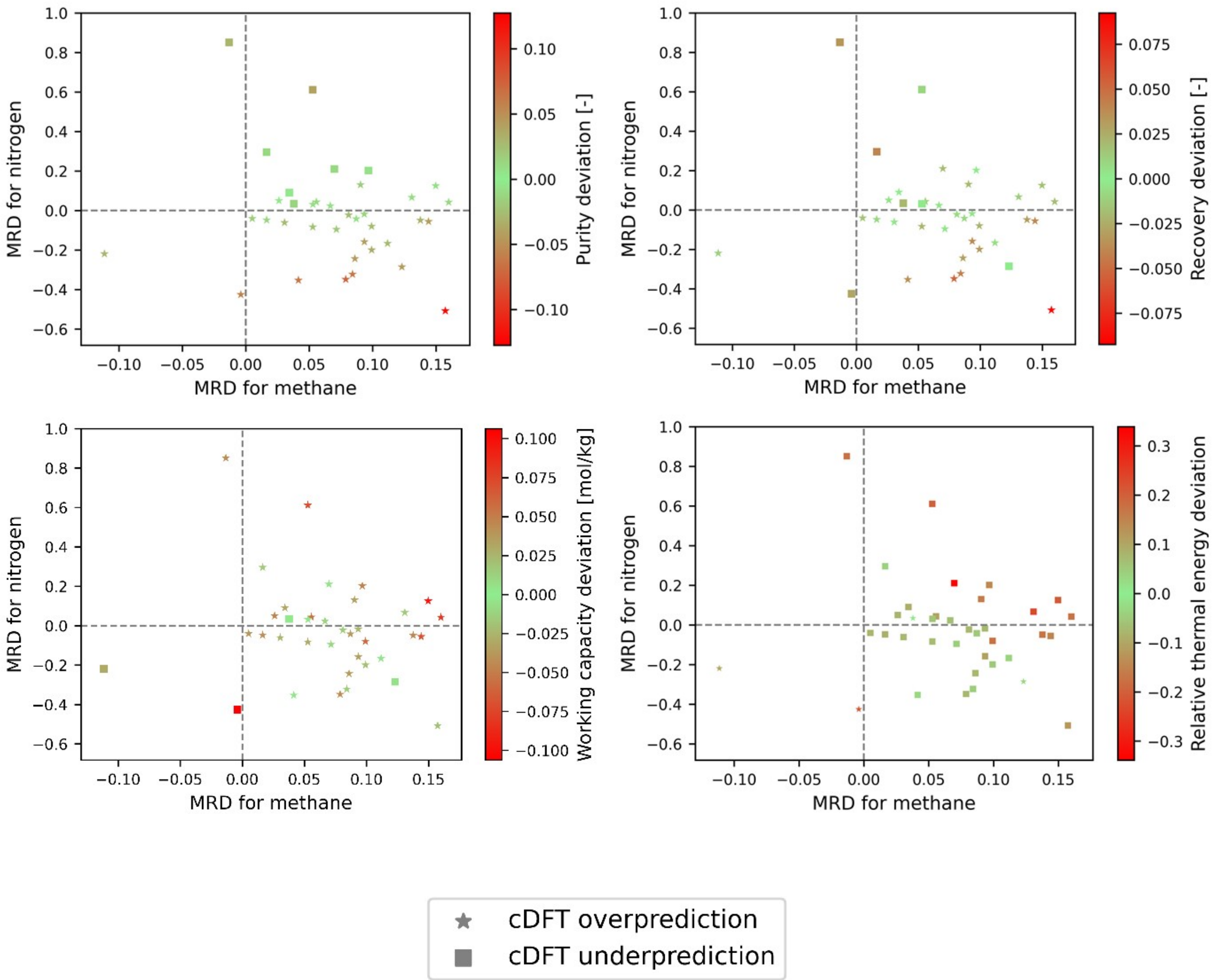

Figure S4: Comparison between the isotherm and process deviation. We compare the mean relative deviation (MRD) between the isotherms predicted by cDFT+CC+IAST and GCMC+CC+IAST of the methane (x-Axis) and nitrogen (y-Axis) isotherms with the predicted product purity, recovery, working capacity, and relative thermal energy deviation (colour code). Positive deviation is indicated as stars, negative deviation as squares. For visibility, we exclude one MOF with a nitrogen MRD of 2.99 and a methane MRD of -0.17.

S7: Process-level screening of CoRE MOF 2025

The calculation time scales with the size of the MOF unit cell. The unit cell size determines how many grid points are required for cDFT to discretize the unit cell. We choose at least 2 grid points per Å in each direction. In Figure S5, we show the calculation time per pressure point as a function of the number of grid points. For smaller unit cells with up to ~130´000 grid points, corresponding to unit cell sizes of ~$(23\ \text{Å})^3$, there is no significant increase in the mean calculation time. For larger unit cells, we observe an increase in calculation time up to a mean calculation time of ~9 s for 2.1 M grid points (Figure S5). Most structures require less than 10 seconds for the cDFT calculation. Large structures with many grid points take on average up to 1 minute per pressure point. We set a grid with a size of at least 2 grid points per Angstrom. Therefore, 1'000'000 grid points correspond to a unit cell size of $(50\text{Å})^3$.

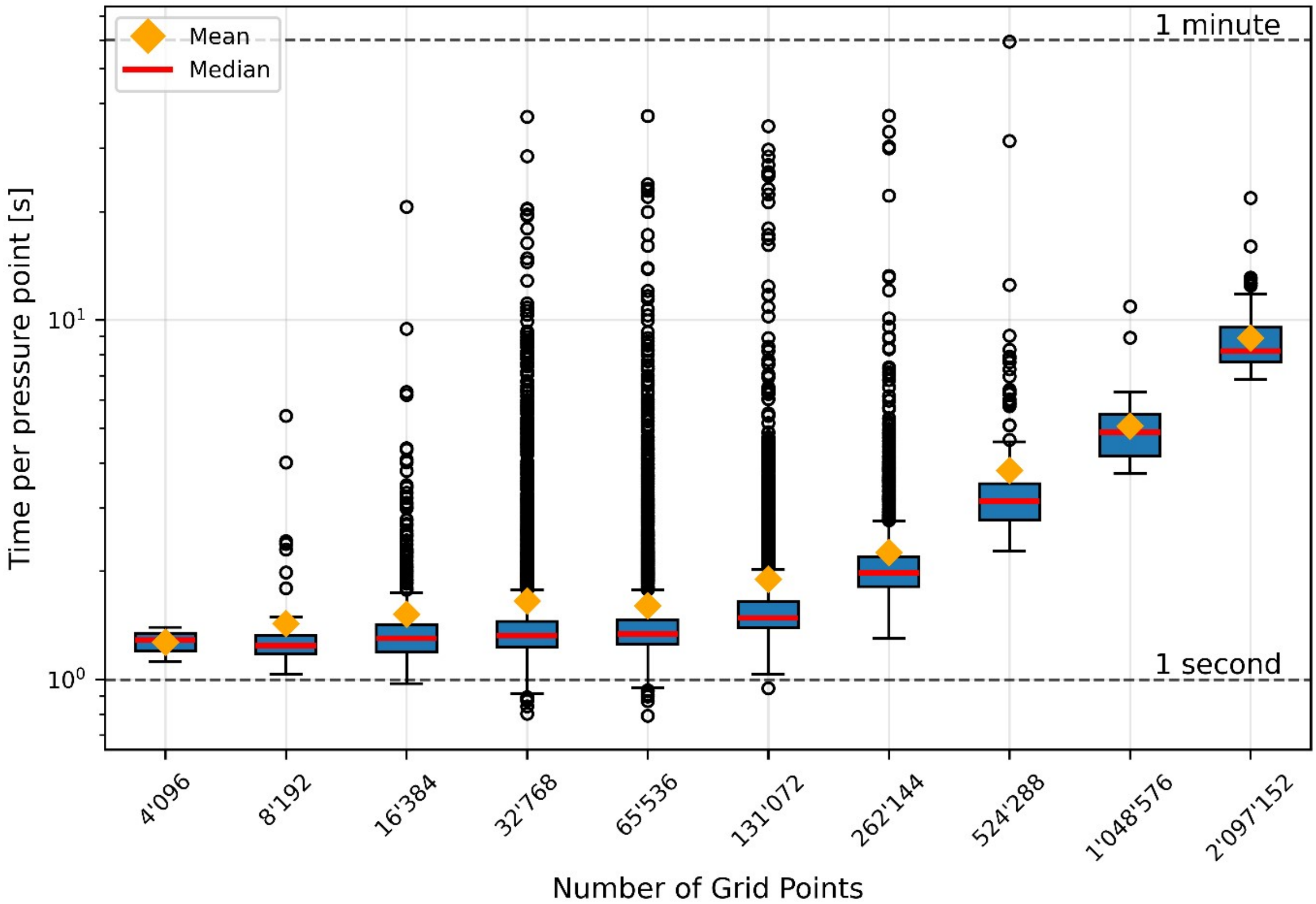

Figure S5: Calculation time per pressure point for each MOF. On the *x*-axis, we show the number of cDFT grid points. The number of grid points correlate with the size of the unit cell. We indicate the median calculation time with a red bar and the mean calculation time with an orange diamond. The calculation time of most MOFs lies within the shown histogram. The outliers are shown as black circles.

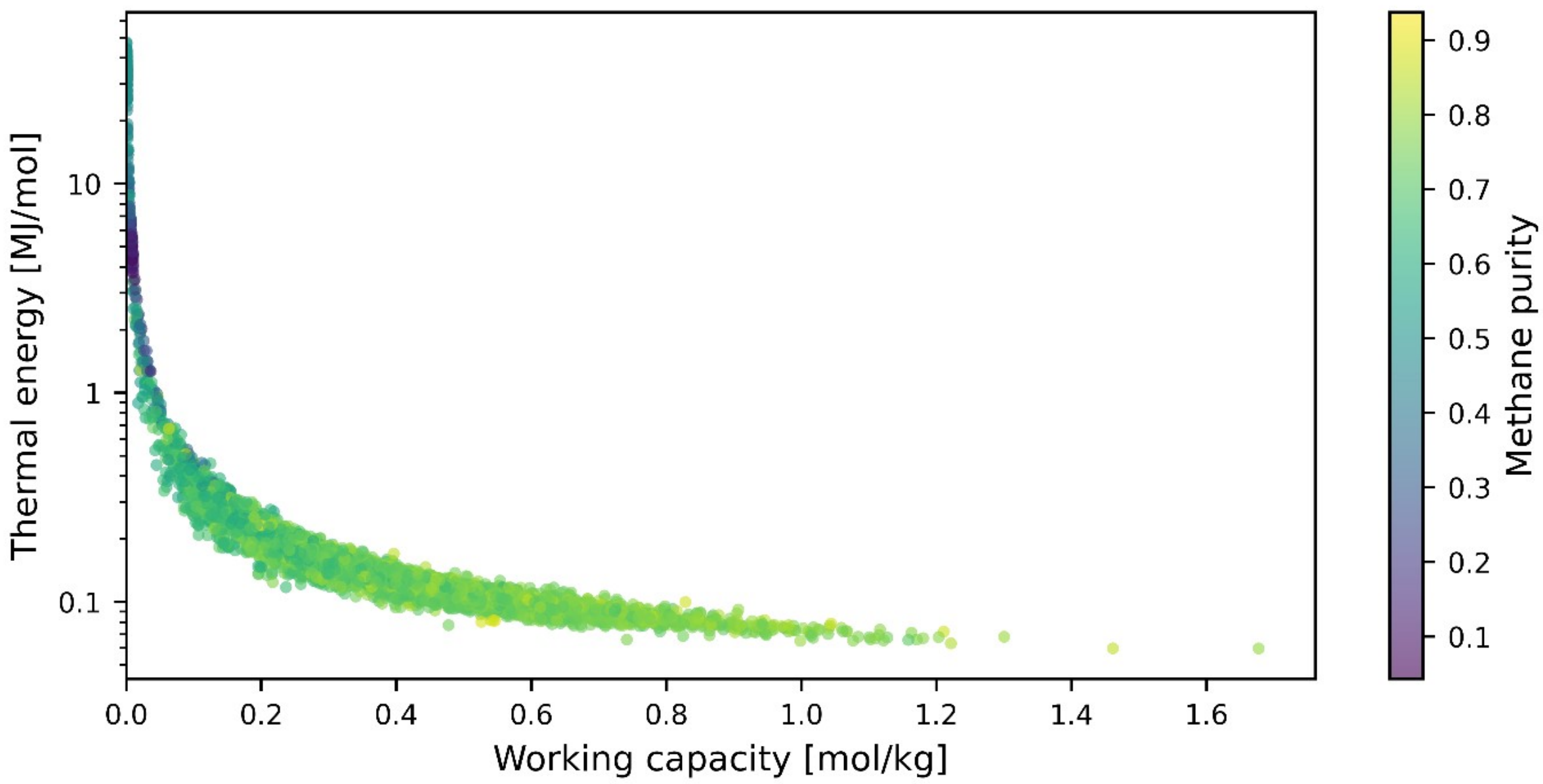

Figure S6: Trade-off between process KPI's. For each structure, we plot the working capacity (*x*-axis) against the thermal energy demand (*y*-axis) and indicate the methane purity as colour. We consider an equimolar methane/nitrogen feed mixture. Thermal energy and working capacity are well-correlated. Promising structures should have a low energy demand and a high working capacity.

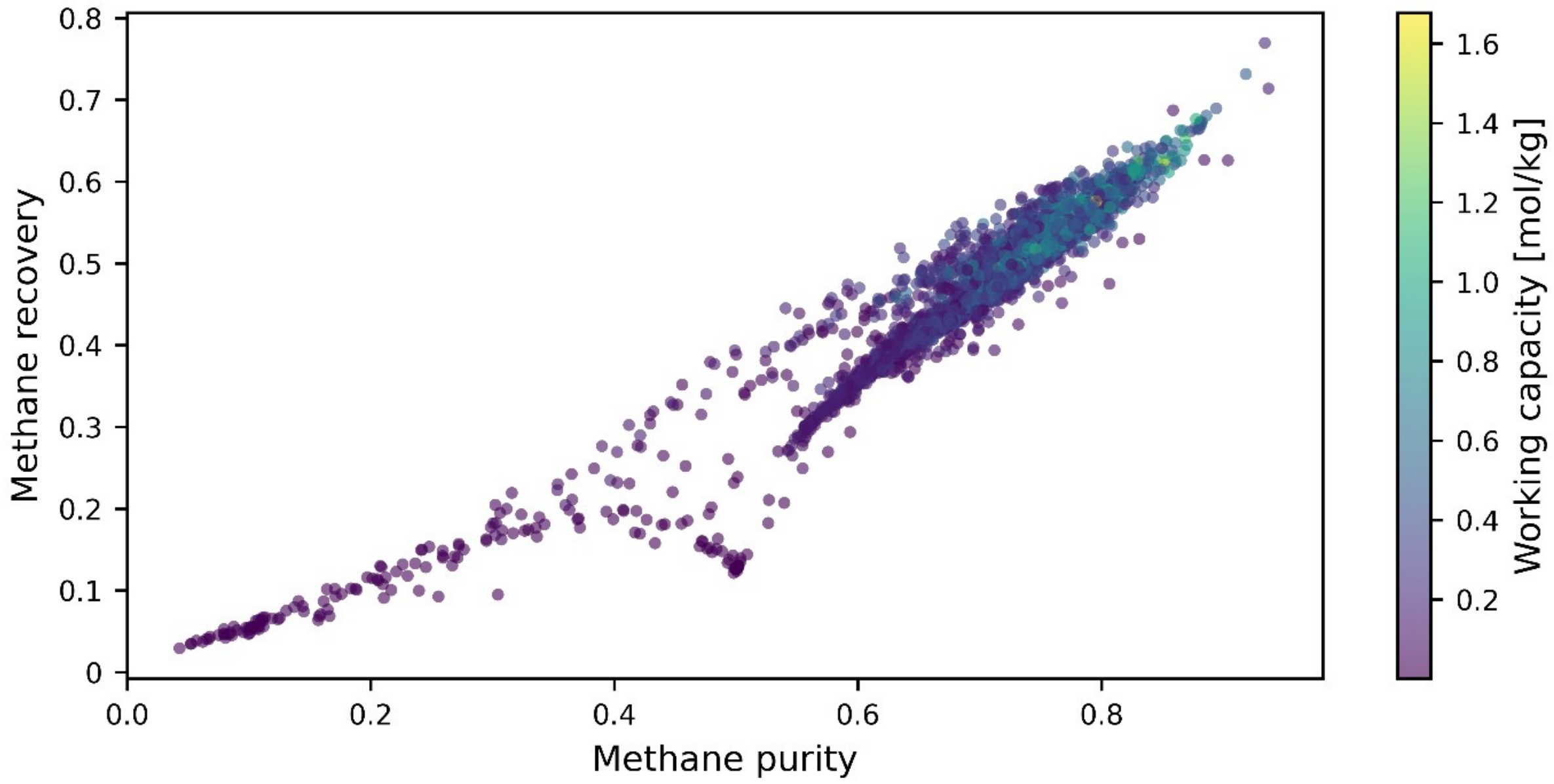

Figure S7: Trade-off between process KPI's. For each structure, we plot the methane purity (*x*-axis) against the methane recovery (*y*-axis) and indicate the working capacity as colour. We consider an equimolar methane/nitrogen feed mixture. Purity and recovery are well-correlated. Promising structures should have a high purity and recovery.

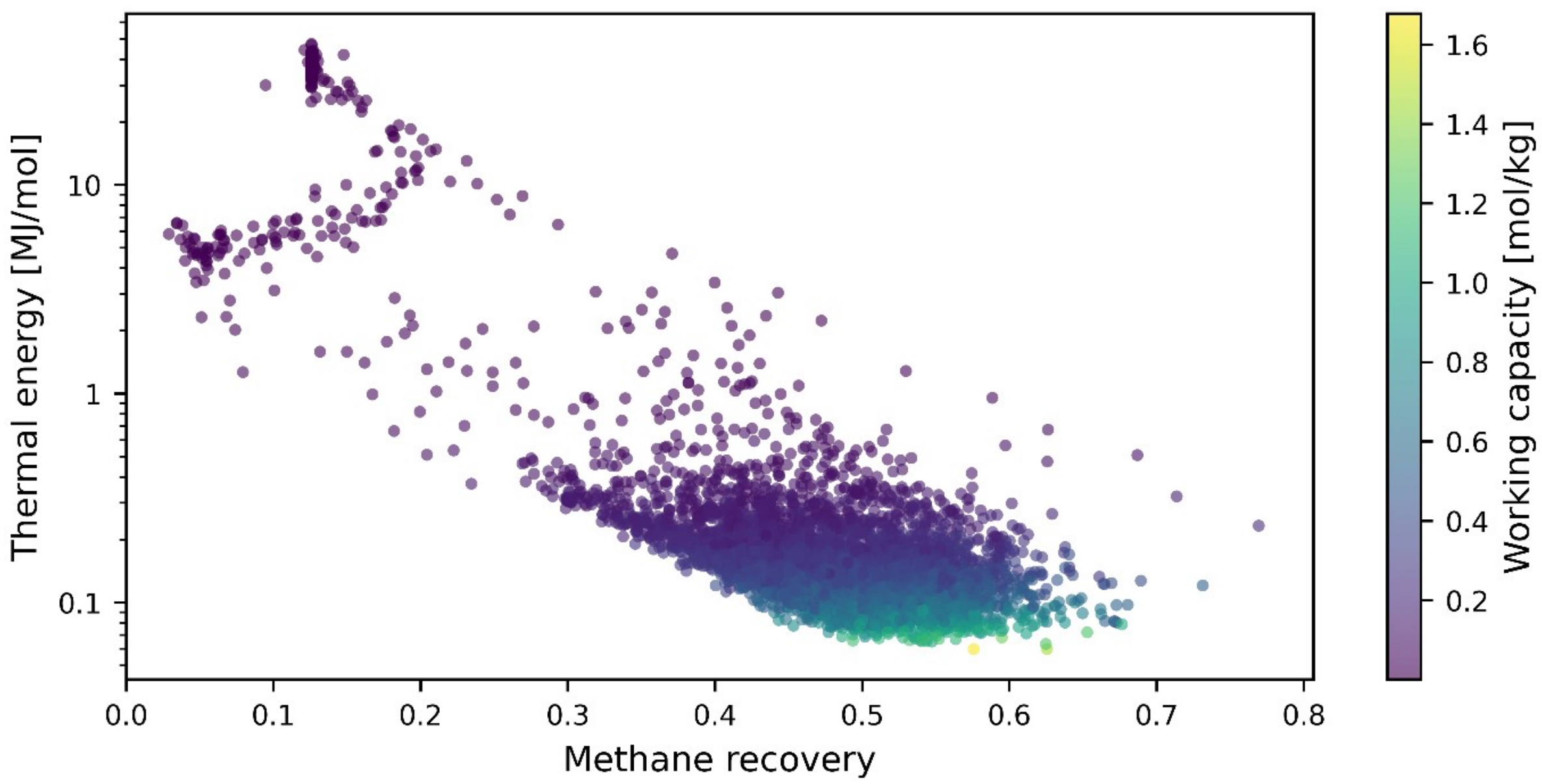

Figure S8: Trade-off between process KPI's. For each structure, we plot the methane recovery (x-axis) against the thermal energy demand (y-axis) and indicate the working capacity as colour. We consider an equimolar methane/nitrogen feed mixture. We observe a trade-off between recovery and thermal energy demand. Promising structures should have a low energy demand and a high recovery.

S8: Process performance across different feed compositions

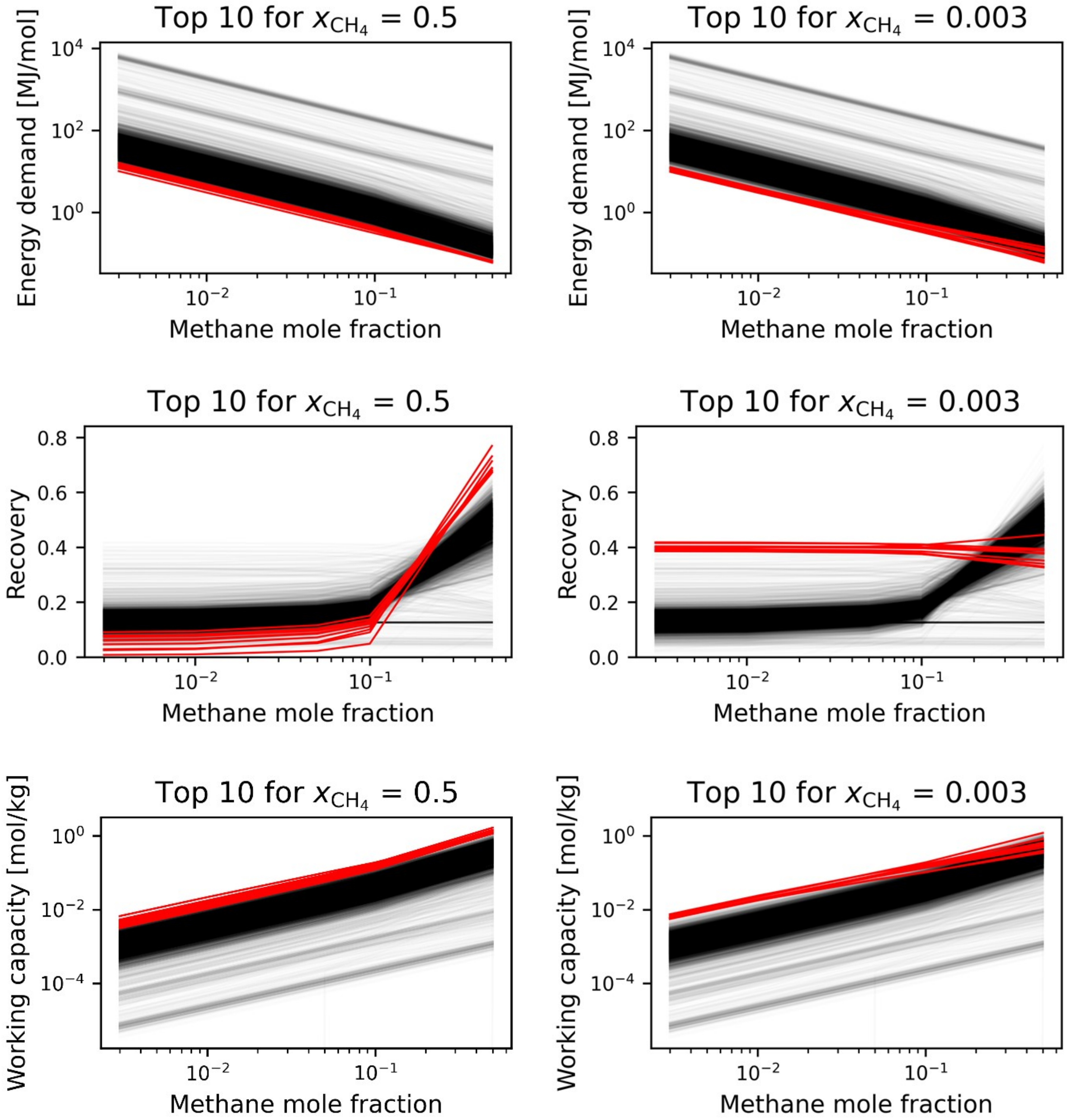

Figure S9: Process performance across different feed compositions. We show the process KPI's energy demand, recovery, and working capacity. We highlight the best 10 structures for the highest methane feed concentration and the lowest feed concentration in red.

S9: Recovery trend for different feed methane mole fractions

In Figure S8, the materials that are the most selective for methane show a high recovery for an equimolar feed. However, the recovery for these materials is strongly dependent on the regeneration during the desorption.

In Figure S9, we show the mole fraction of the gas phase during the desorption and cooling for different feed compositions and desorption temperatures. The MOF FALQIU shows the highest recovery for an equimolar feed and is very selective for methane. For an equimolar feed, the MOF already regenerates at a desorption temperature of 348.15 K, which is indicated by the mole fraction of methane in the gas phase approaching 1 at the end of the desorption.

During the cooling step, additional feed is fed into the column to maintain a pressure of 1 bar. If the column is regenerated, most of the methane in the feed stream is adsorbed and mostly nitrogen remains in the gas phase, resulting in a low methane mole fraction in the gas phase. During the subsequent adsorption step, some of the gas phase is pushed out of the column by the feed stream and lost as waste stream. Therefore, a lower methane concentration in the gas phase after the cooling yields a higher recovery, as less methane is lost as waste stream during the adsorption step.

When the desorption temperature for FALQIU is increased to 448.15 K, the recovery increases, but not significantly, compared to the jump from a 50 K to a 150 K temperature swing.

However, for a methane feed concentration of 0.1 and a desorption temperature of 348.15 K, the MOF FALQIU does not regenerate. Consequently, the column still contains methane and nitrogen in an equilibrium, and the cooling trajectory follows almost exactly the desorption line. After the cooling, methane remains in the gas phase and is lost as waste stream, impeding the recovery.

A regeneration of the adsorbent is achieved for a higher desorption temperature of 448.15 K. Consequently, the recovery increases significantly for the higher desorption temperature and a methane feed concentration of 0.1.

On the other hand, some MOFs in Figure S8 show a constant recovery over all feed concentrations. These MOFs are not selective for methane. The product has the same composition as the feed stream. The product stream results from the desorption and volume change of the gas phase.

In Figure S10, we show a detailed plot of the recovery as a function of the methane feed concentration for two desorption temperatures and three different structures. FALQIU is the MOF which is very selective for methane and shows the highest recovery at high methane feed concentrations. NIRPAI is less selective for methane, with a lower recovery at high feed concentrations. VELVEO is not selective for methane. This MOF does not purify methane, i.e., the product stream has a similar composition as the feed stream. The recovery is just a result of the volume exchange during desorption and cooling, as explained before.

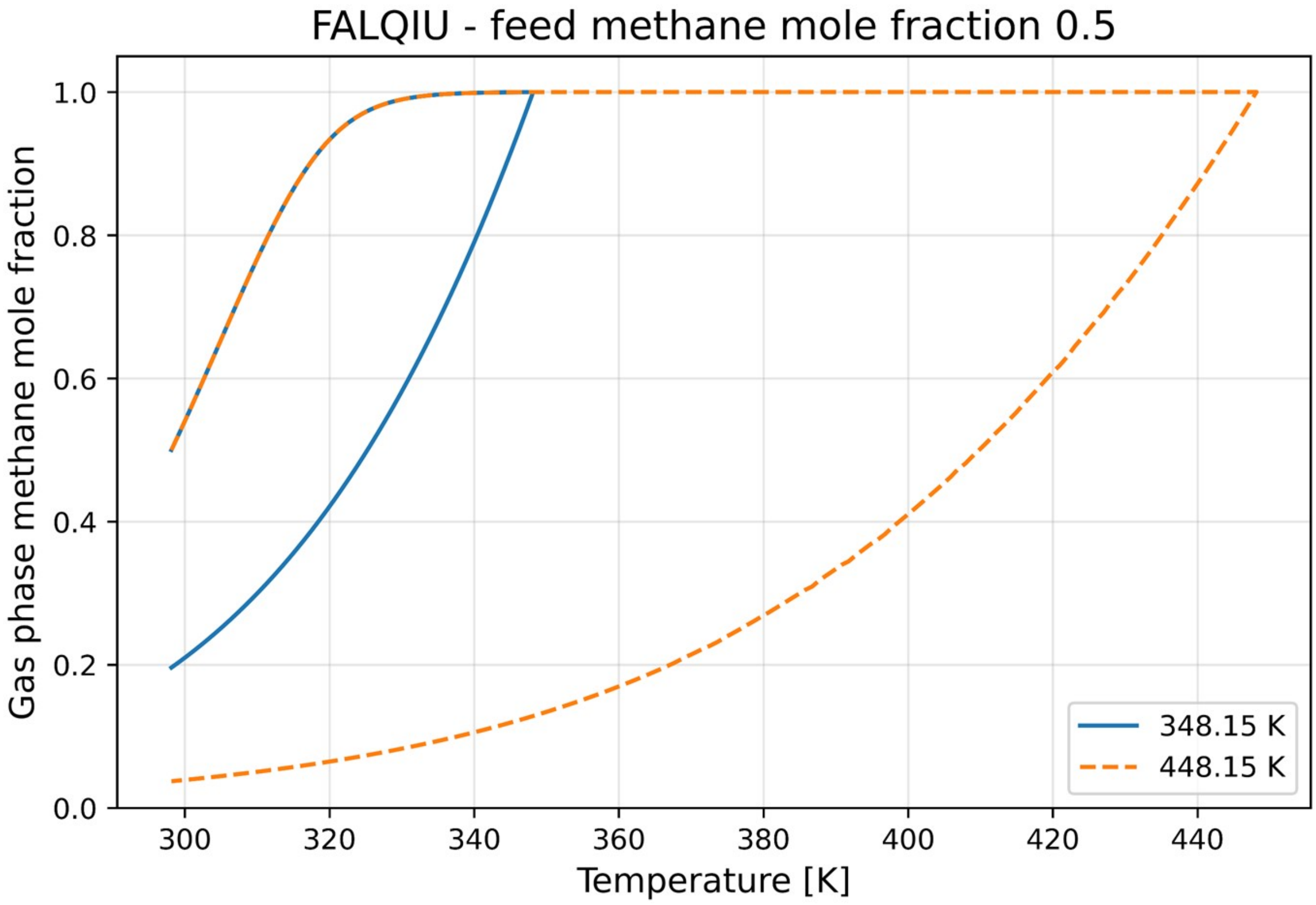

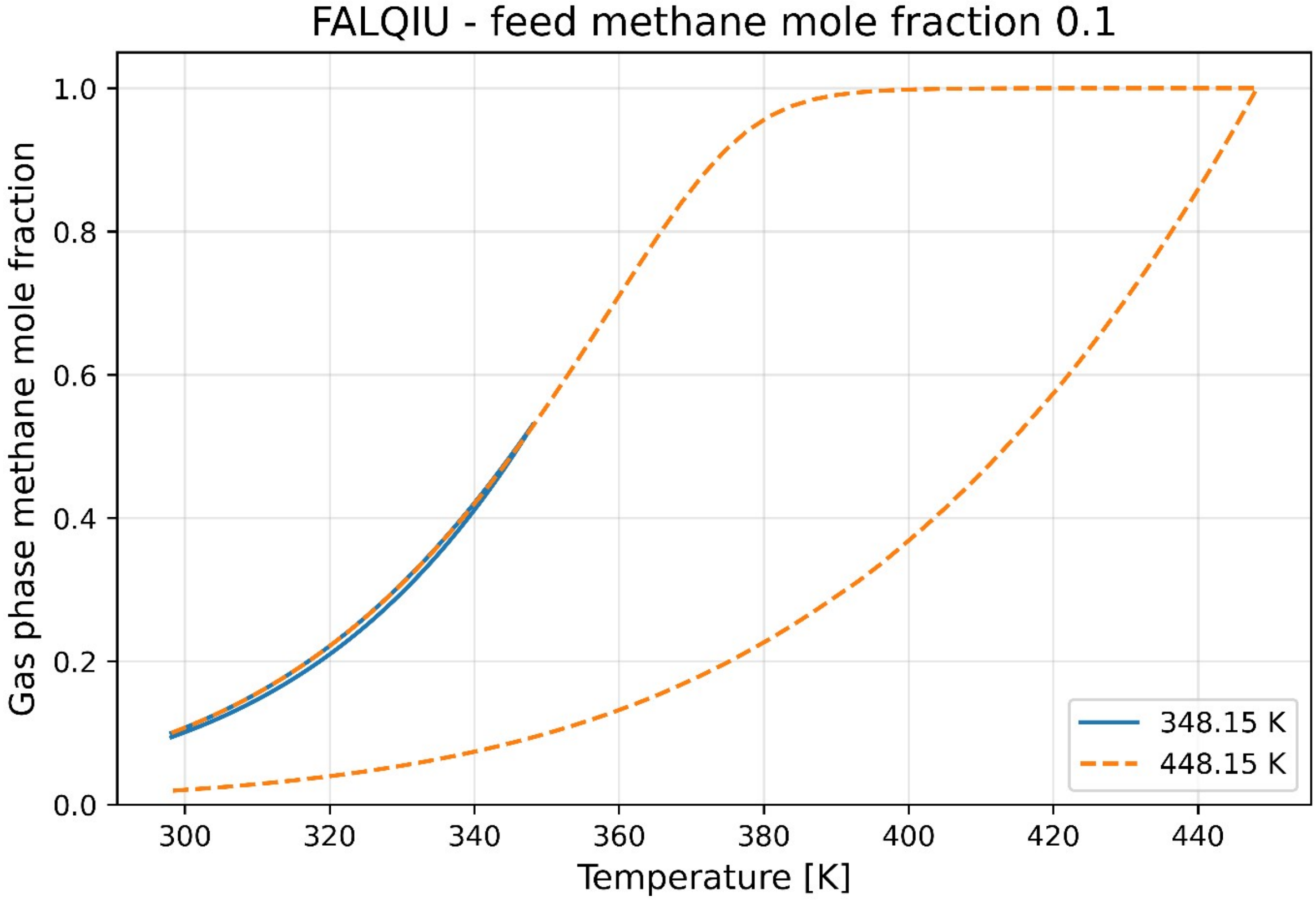

Figure S10: Gas phase concentration of methane over the temperature swing for the structure FALQIU. We compare two different feed methane mole fractions (0.1 and 0.5) and different desorption temperatures (348.15 K and 448.15 K).

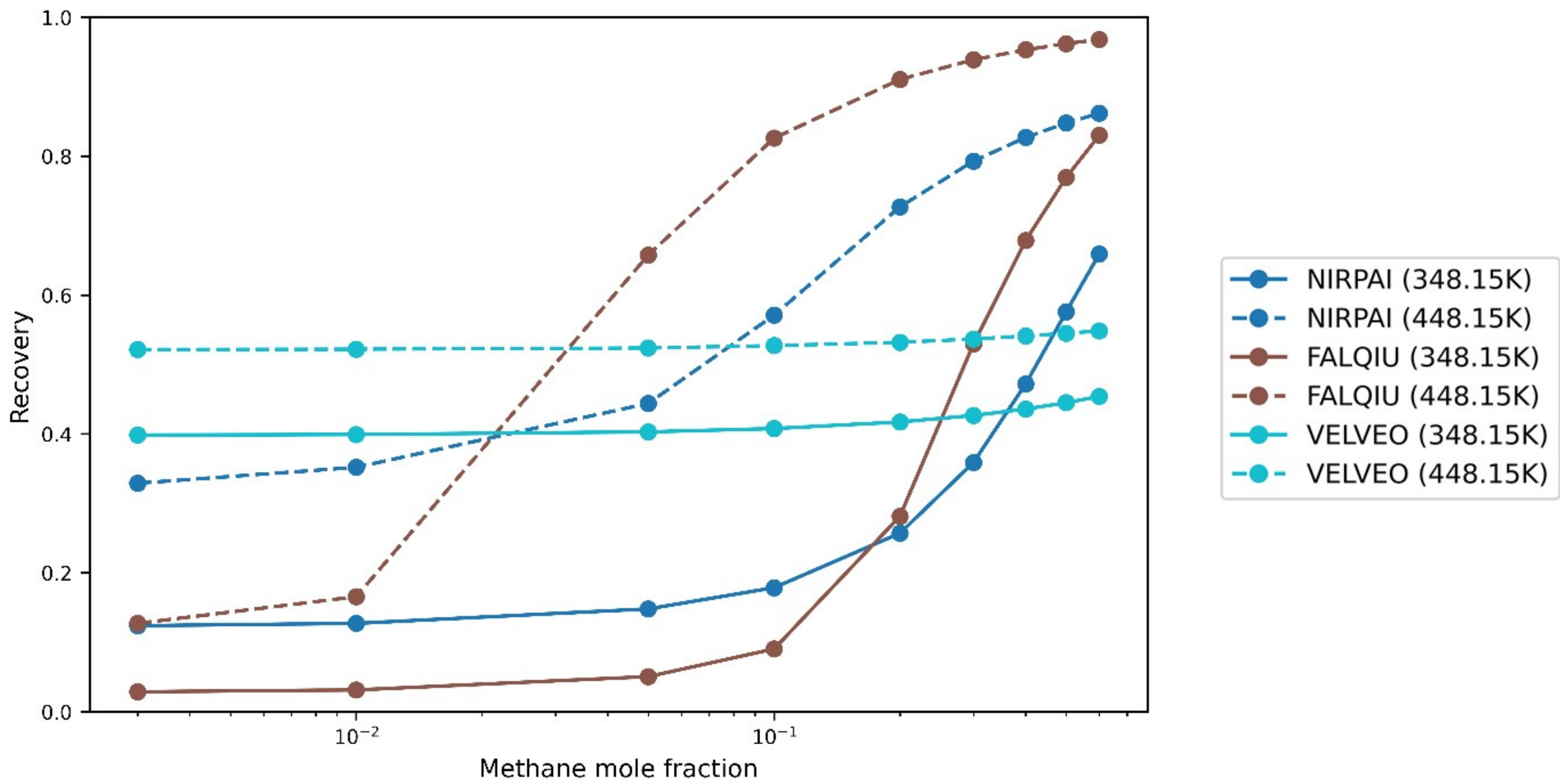

Figure S11: Methane recovery over the methane feed concentration for different structures and desorption temperatures.

S10: Process performance at higher desorption temperatures

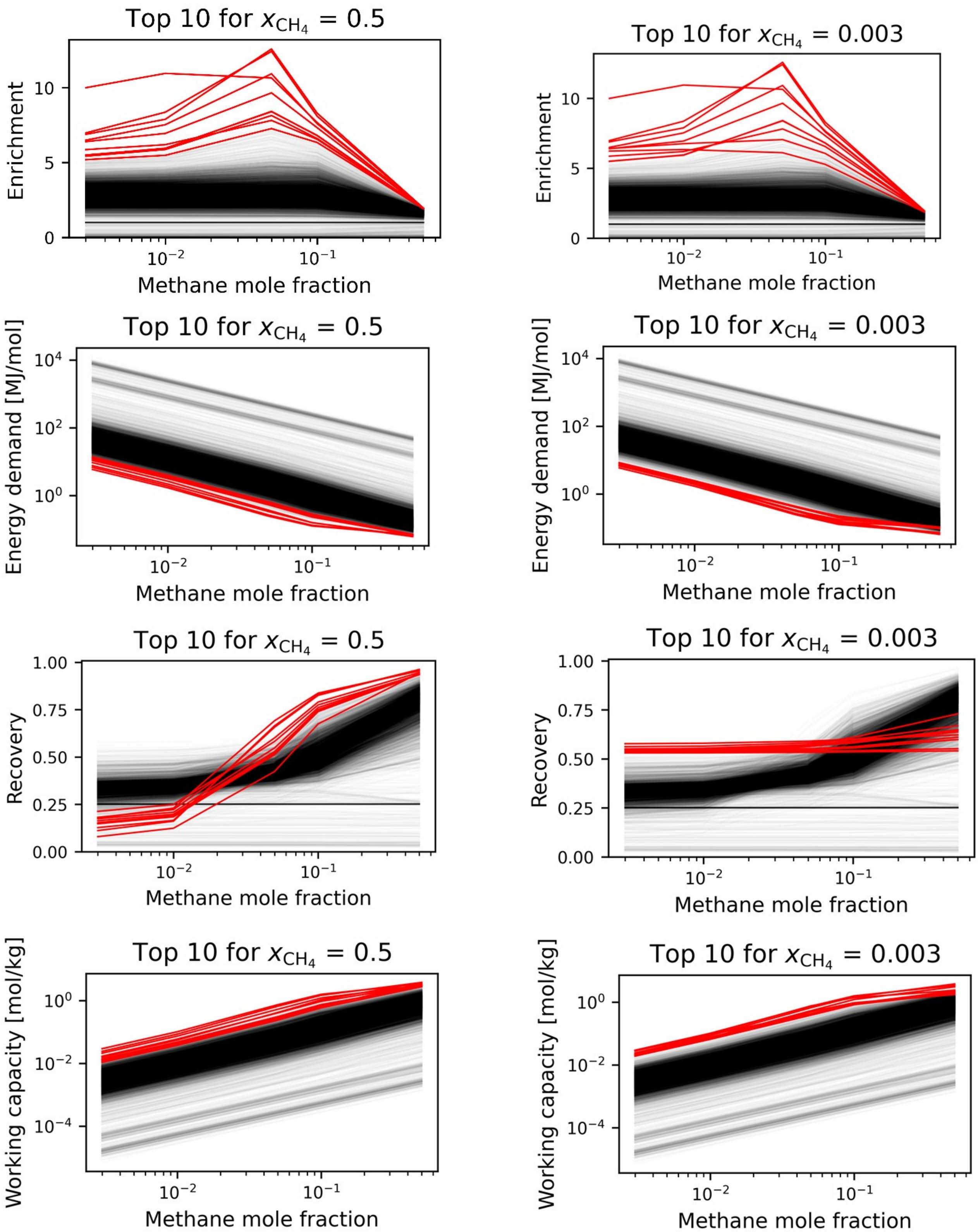

Figure S12: Process performance across different feed compositions at a desorption temperature of T_des=448.15 K.

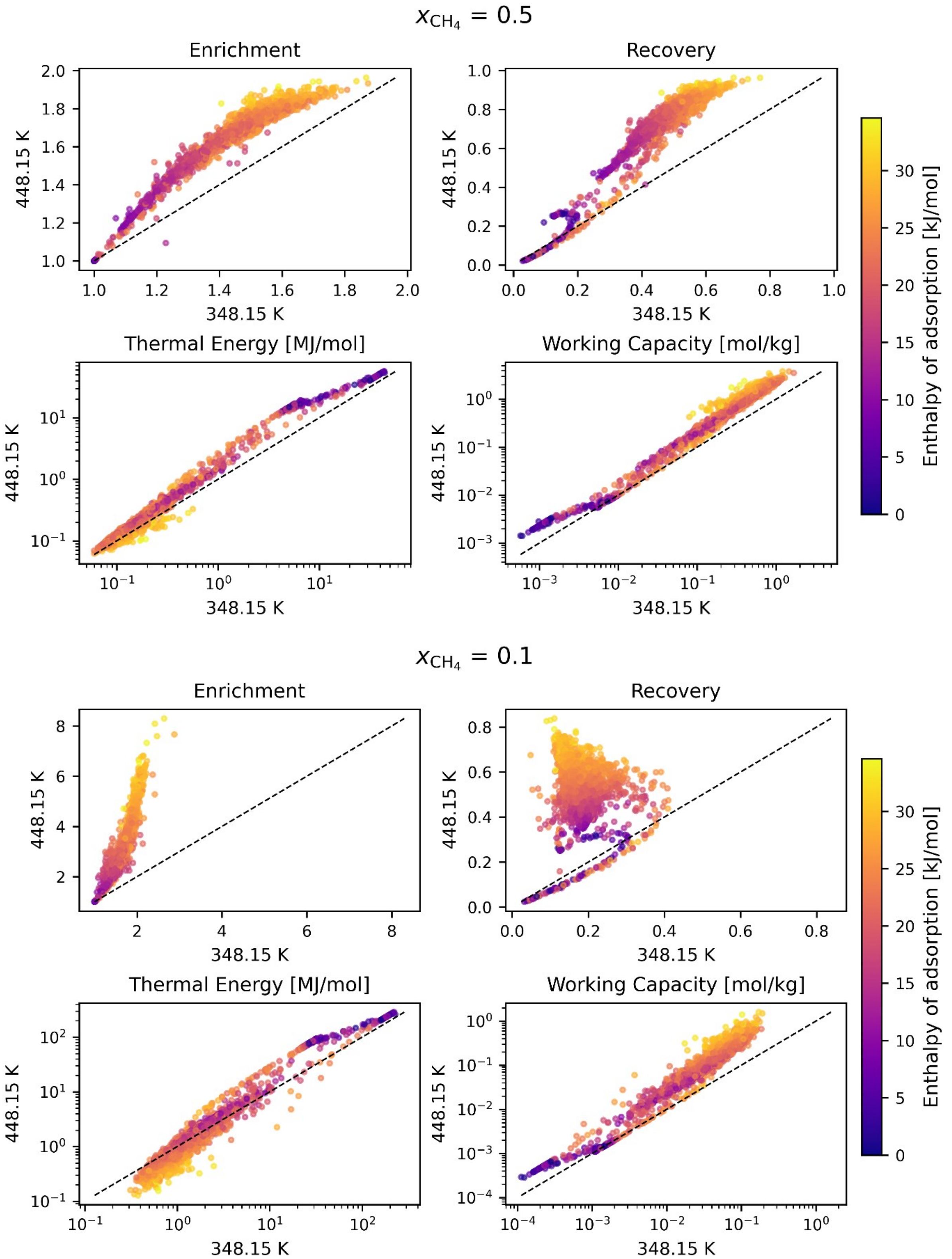

Figure S13: Comparison of the process performance for the two different desorption temperatures $T_{des} = 348.15\ K$ and $T_{des} = 448.15\ K$. Additionally, we indicate the enthalpy of adsorption as colour.

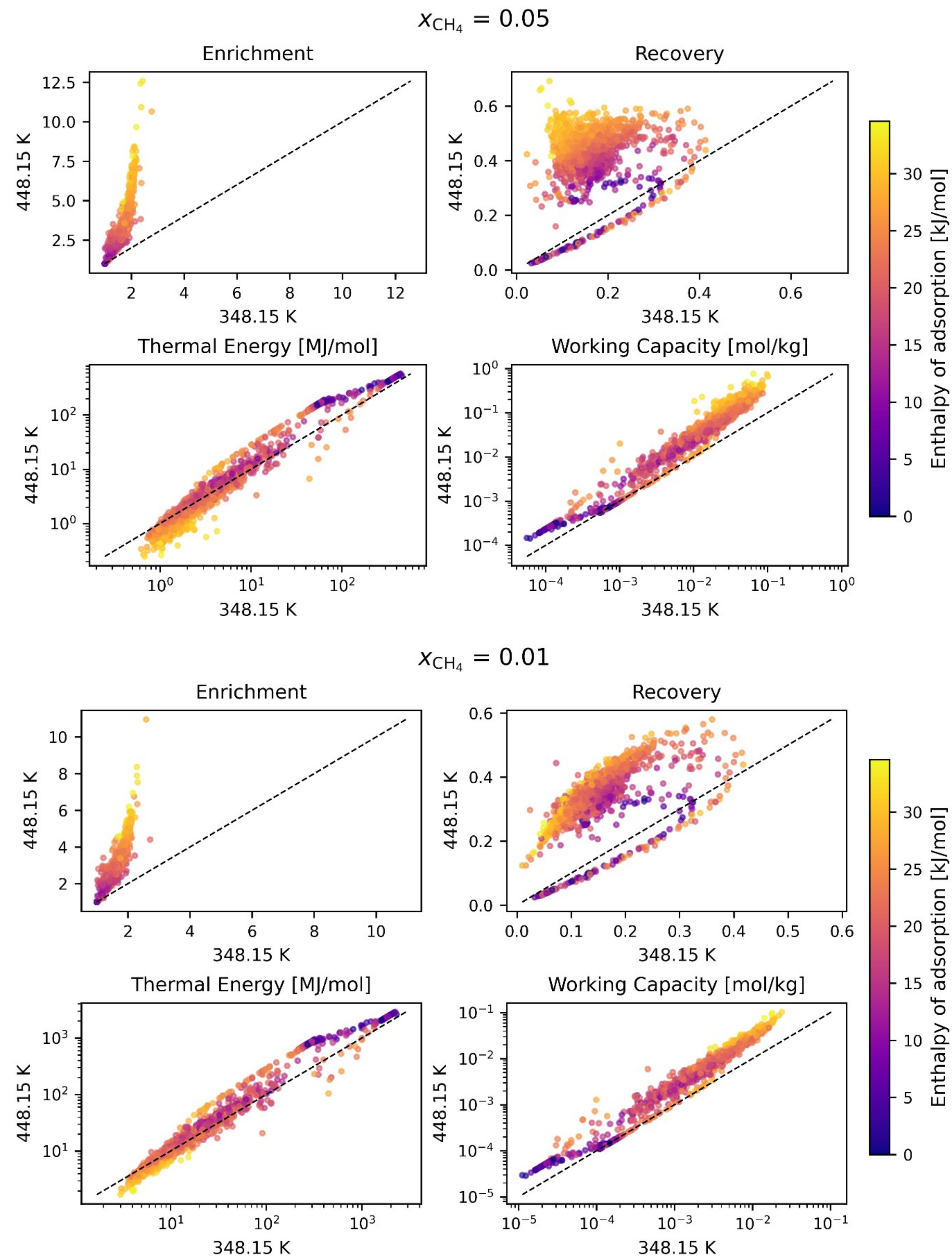

Figure S13: Continuation from the previous page.

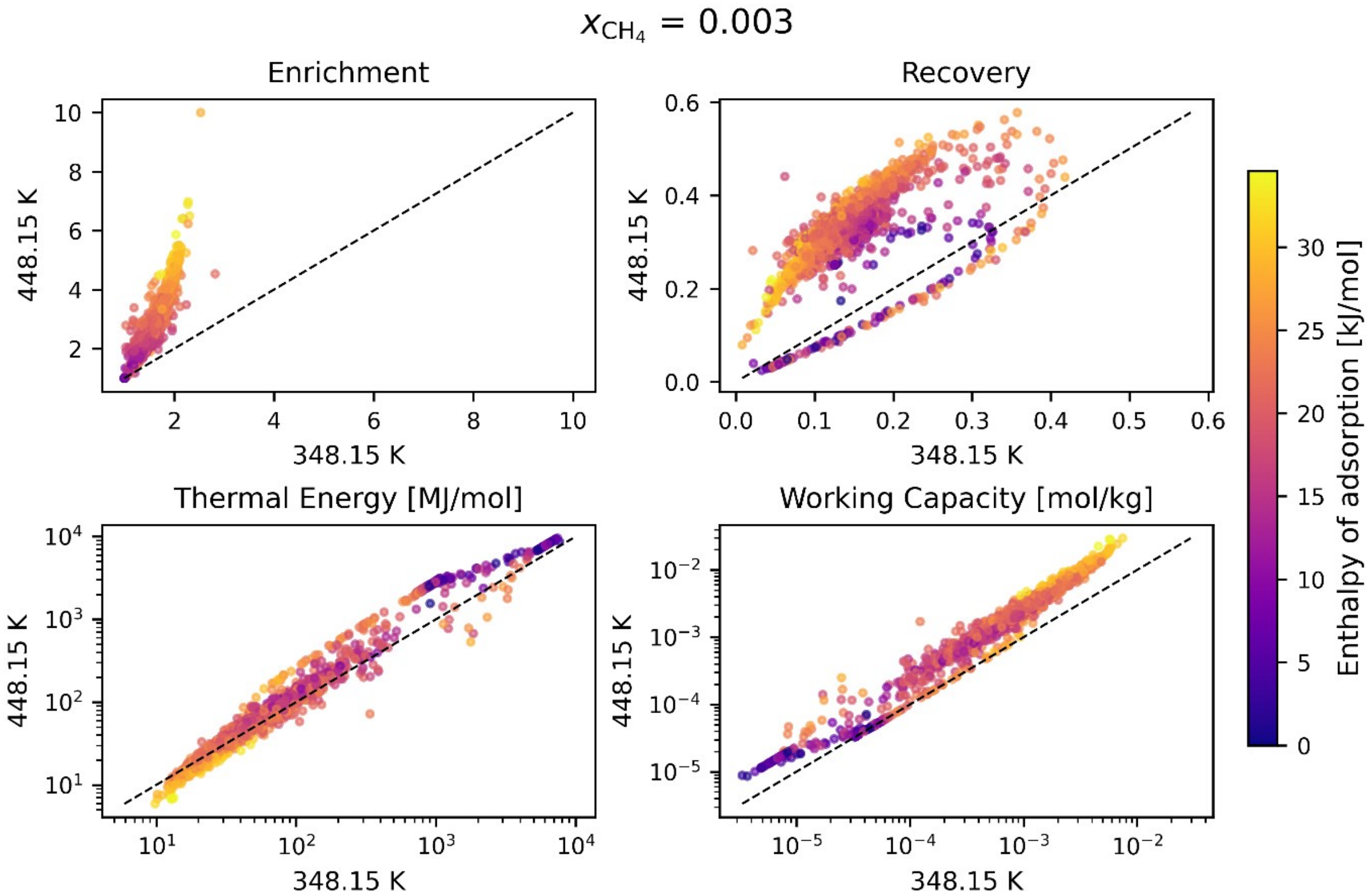

Figure S13: Continuation from the previous page.

In the previous Section S9, we show that higher desorption temperature is required to achieve regeneration of the MOFs that are strongly selective for methane. The trajectory of the recovery in Figure S12 indicates that still not all MOFs regenerate at the higher desorption temperature of 448.15 K across all feed concentrations. In practice, the feed concentration is defined by the application and the desorption temperature by the availability of heat sources. Therefore, it is necessary to evaluate which MOFs are suitable for which application.

Increasing the desorption temperature further presents the additional challenge that MOFs can decompose at higher temperatures.[5] Nevertheless, the selective MOFs still indicate superior performance compared to the other MOFs in terms of enrichment, working capacity, and energy demand.

S11: Isotherm validation for promising MOFs

In Figure S11, we show the isotherms for the 5 promising structures that were identified in the screening. For each structure, we run binary GCMC simulations of an equimolar methane/nitrogen mixture at the pressures 0.1 bar, 1 bar, and 10 bar and the temperatures 298.15 K and 348.15 K.

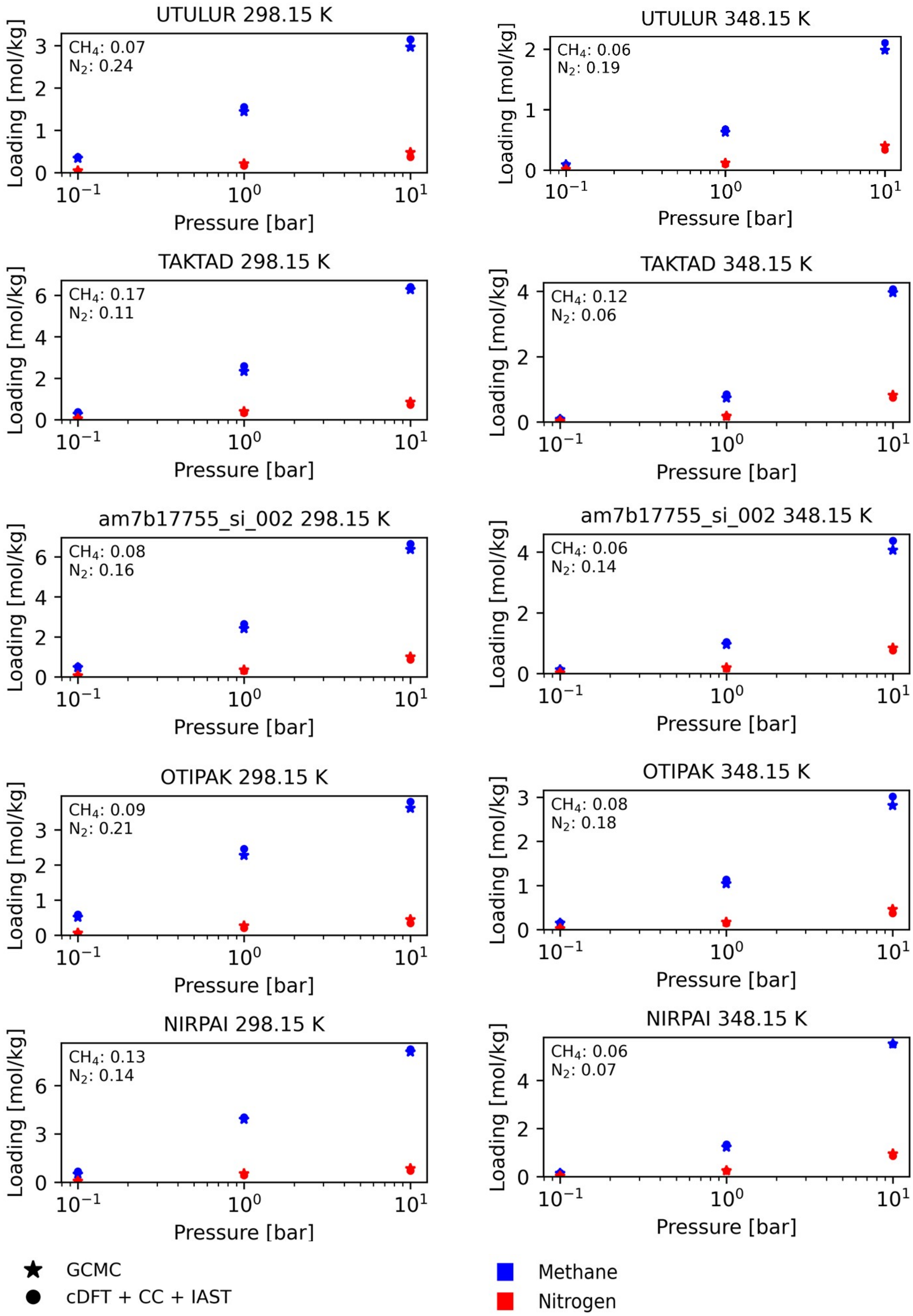

Figure S14: Isotherm validation for promising MOFs. We compare isotherms predicted from classical density functional theory + Clausius-Clapeyron temperature extrapolation + ideal adsorbed solution theory with binary grand-canonical Monte Carlo simulations at two temperatures, 298.15K and 348.15K, respectively. We indicate the isotherm MARD in the upper left corner.

S12: Sensitivity analysis of process parameters

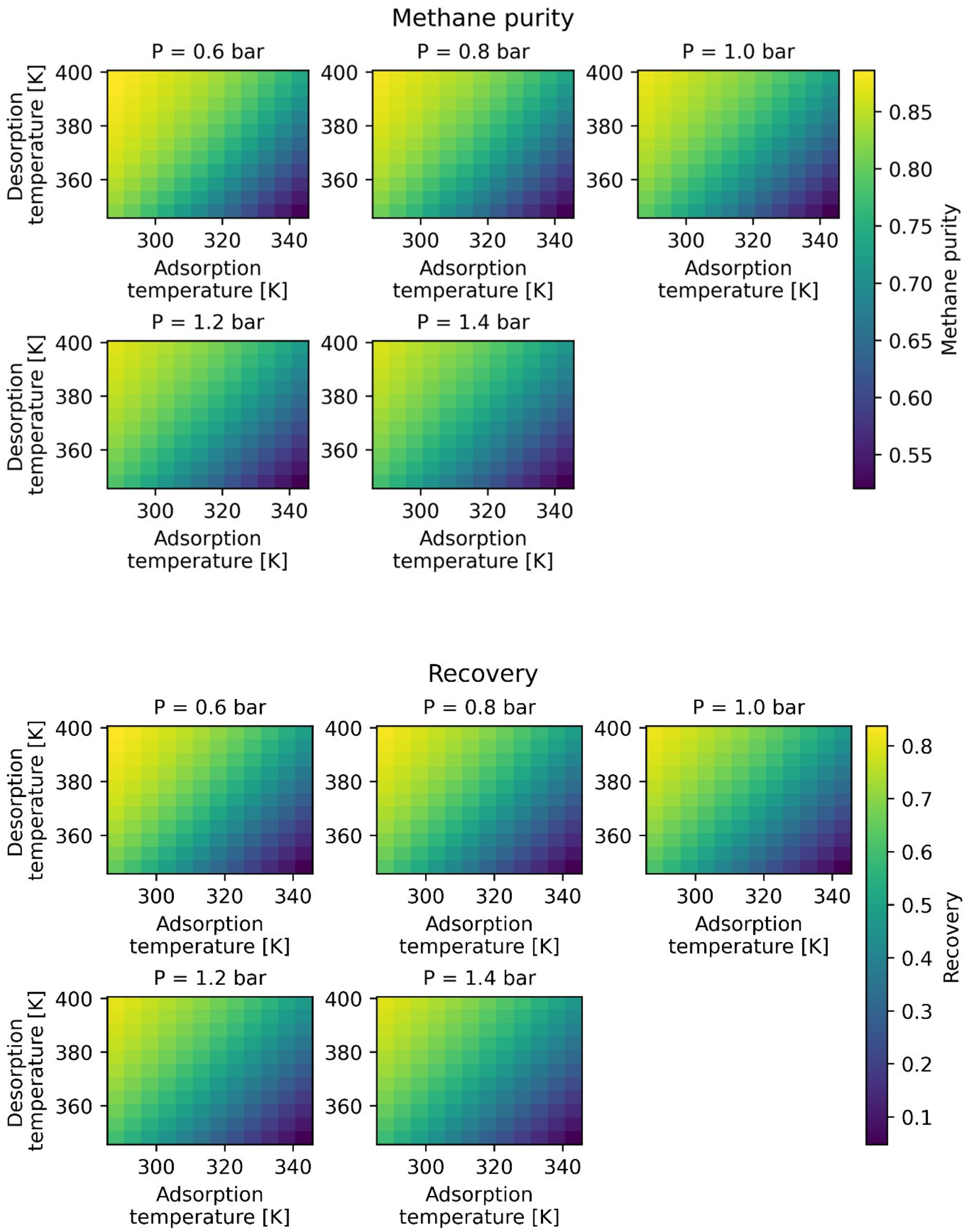

Figure S15: Process parameter screening for the MOF UTULUR. We evaluate the process performance across different adsorption and desorption temperatures, as well as different operating pressures. Low adsorption and high desorption temperatures are beneficial for all process KPI's. The process operation at low pressure yields higher purity and higher recovery, however, it also reduces the working capacity and increases the specific thermal energy demand.

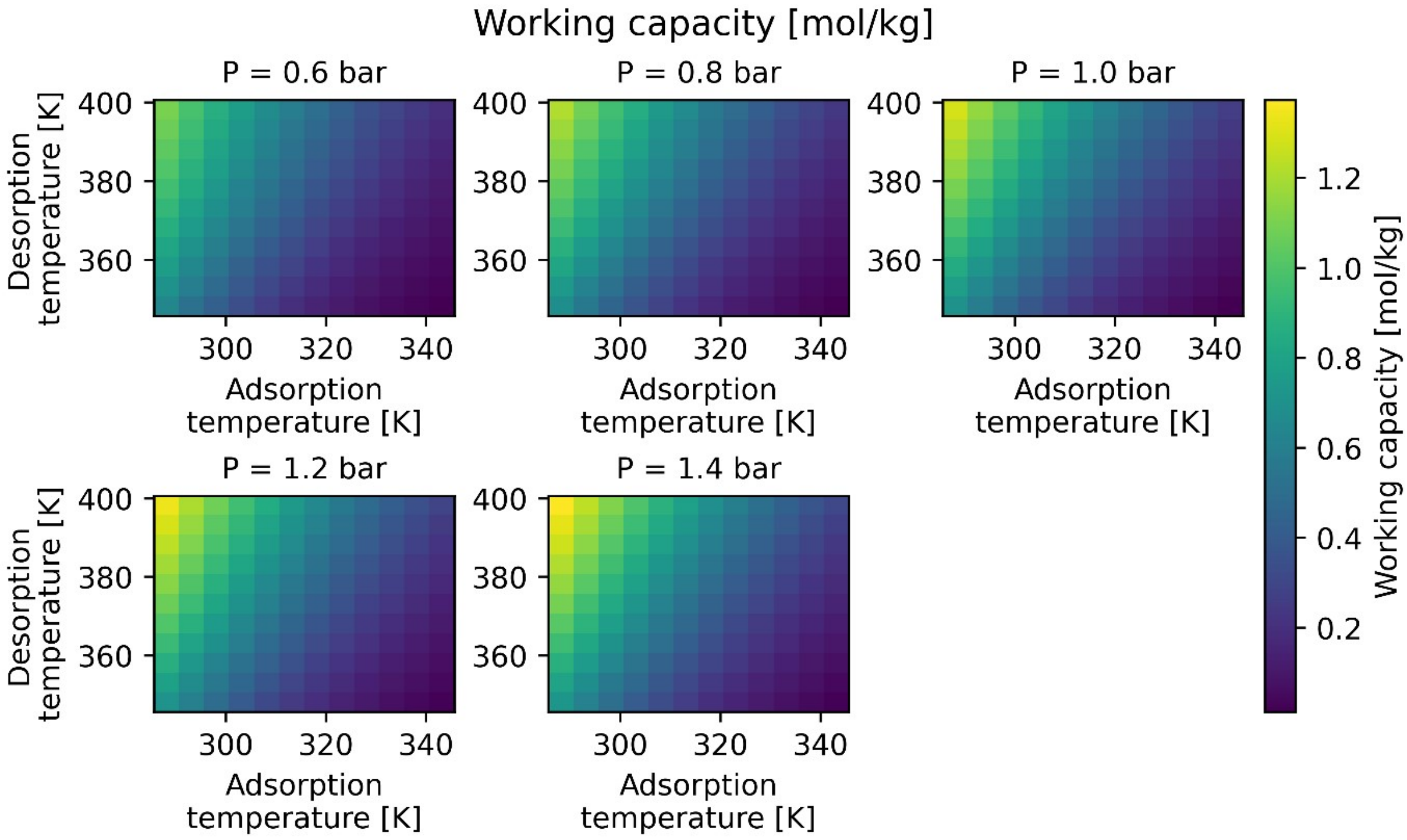

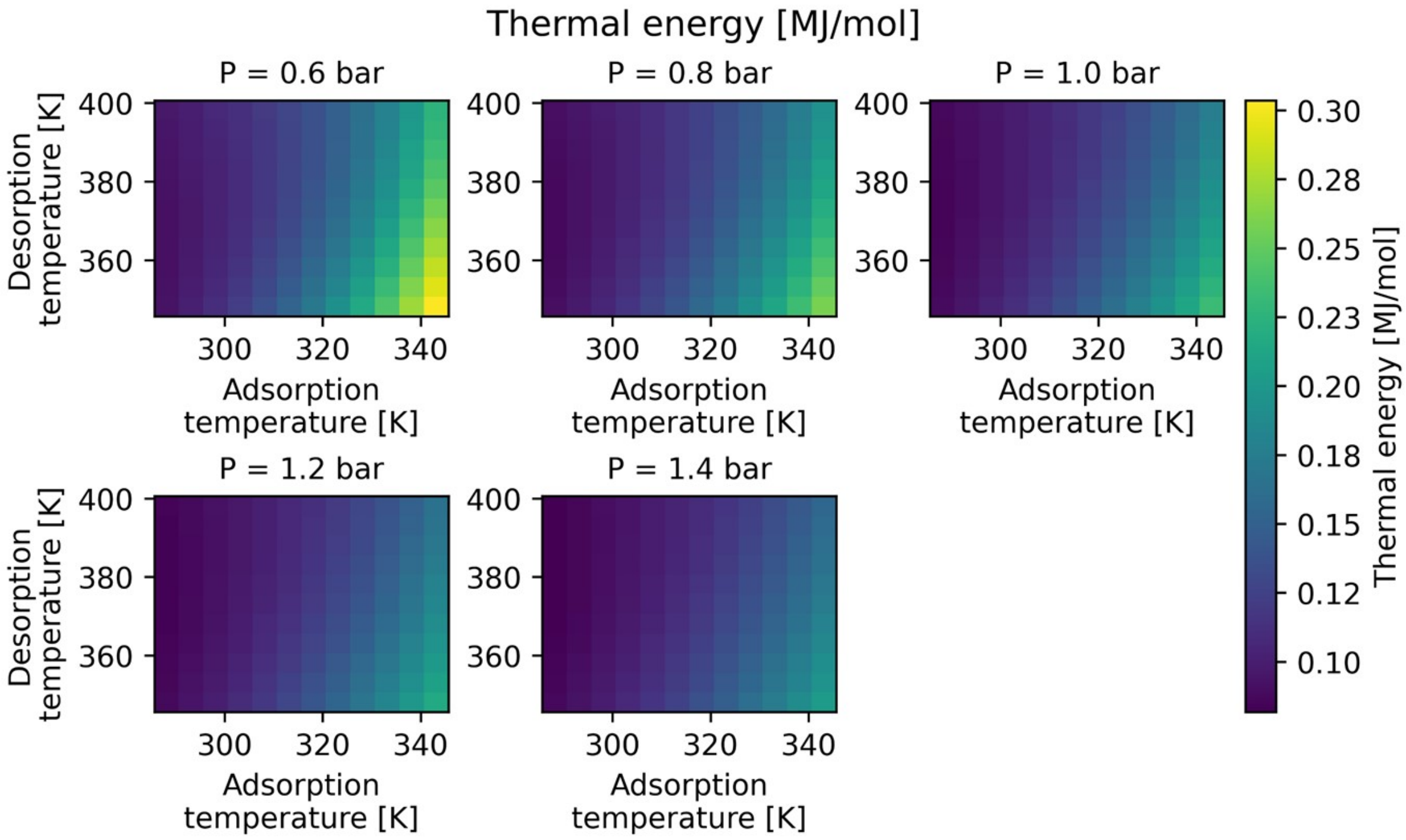

Figure S15: Continuation from the previous page.

1. A. Ajenifuja, L. Joss and M. Jobson, *Ind. Eng. Chem. Res.*, 2020, **59**, 3485–3497.
2. S. M. Moosavi, B. A. Novotny, D. Ongari, E. Moubarak, M. Asgari, O. Kadioglu, C. Charalambous, A. Ortega-Guerrero, A. H. Farmahini, L. Sarkisov, S. Garcia, F. Noe and B. Smit, *Nat. Mater.*, 2022, **21**, 1419–1425.
3. C. Charalambous, E. Moubarak, J. Schilling, E. Sanchez Fernandez, J. Y. Wang, L. Herraiz, F. McIlwaine, S. B. Peh, M. Garvin, K. M. Jablonka, S. M. Moosavi, J. Van Herck, A. Y. Ozturk, A. Pourghaderi, A. Y. Song, G. Mouchaham, C. Serre, J. A. Reimer, A. Bardow, B. Smit and S. Garcia, *Nature*, 2024, **632**, 89–94.
4. R. Stierle, G. Bauer, N. Thiele, B. Bursik, P. Rehner and J. Gross, *Chem. Eng. Sci.*, 2024, **298**.
5. A. Nandy, G. Terrones, N. Arunachalam, C. Duan, D. W. Kastner and H. J. Kulik, *Sci. Data*, 2022, **9**, 74.
